# Removal of Trace Organic Contaminants by Parallel Operation of Reverse Osmosis and Granular Activated Carbon for Drinking Water Treatment

**DOI:** 10.3390/membranes11010033

**Published:** 2021-01-02

**Authors:** Norbert Konradt, Jan Gerrit Kuhlen, Hans-Peter Rohns, Birgitt Schmitt, Uwe Fischer, Timo Binder, Vera Schumacher, Christoph Wagner, Stefan Kamphausen, Uwe Müller, Frank Sacher, Peter Janknecht, Ralph Hobby, Ibrahim M. A. ElSherbiny, Stefan Panglisch

**Affiliations:** 1 Department of Waterworks, Stadtwerke Düsseldorf AG, Wiedfeld 50, 40589 Düsseldorf, Germany; hprohns@swd-ag.de (H.-P.R.); bschmitt@swd-ag.de (B.S.); ufischer@swd-ag.de (U.F.); tbinder@swd-ag.de (T.B.); cwagner@swd-ag.de (C.W.); skamphausen@swd-ag.de (S.K.); 2 Viega Technology GmbH & Co. KG, Viegaplatz 1, 57439 Attendorn, Germany; Jan-Gerrit.Kuhlen@viega.de; 3 Berliner Wasserbetriebe, Motardstraße 35, 13629 Berlin, Germany; vera.schumacher@bwb.de; 4 DVGW-Technologiezentrum Wasser, Karlsruher Straße 84, 76139 Karlsruhe, Germany; frank.sacher@tzw.de; 5 Enercity Netz GmbH, Auf der Papenburg 18, 30459 Hannover, Germany; peter.janknecht@enercity-netz.de; 6 Chair for Mechanical Process Engineering and Water Technology, University of Duisburg-Essen, 47057 Duisburg, Germany; ralph.hobby@uni-due.de (R.H.); ibrahim.elsherbiny@uni-due.de (I.M.A.E.); stefan.panglisch@uni-due.de (S.P.)

**Keywords:** drinking water treatment, low-pressure reverse osmosis, activated carbon filtration, trace organic contaminants, membrane fouling

## Abstract

In response to increasingly stringent restrictions for drinking water quality, a parallel operation of two common technologies, low-pressure reverse osmosis (LPRO) and activated carbon filtration (ACF), was investigated in a comprehensive five-month pilot study for the removal of 32 typical trace organic contaminants (TrOCs) from Rhine bank filtrates employing a semi- technical plant. TrOCs have been divided into three groups: polyfluorinated aliphatic compounds; pharmaceuticals, pesticides and metabolites; in addition to volatiles, nitrosamines and aminopolycarboxylic acids, which were also examined. The net pressure behavior, normalized salt passage and rejection of TrOCs by LPRO were investigated and compared with ACF operation. In addition, autopsies from the leading and last membrane modules were performed using adenosine triphosphate (ATP), total organic carbon (TOC), ICP-OES and SEM-EDX techniques. Generally, rather stable LPRO membrane performance with limited membrane fouling was observed. TrOCs with a molecular weight of ≥ 150 Da were completely retained by LPRO, while the rejection of di- and trichloro compounds improved as the filtration progressed. ACF also showed significant removal for most of the TrOCs, but without desalination. Accordingly, the ACF and LPRO can be operated in parallel such that the LPRO permeate and the ACF-treated bypass can be mixed to produce drinking water with adjustable hardness and significantly reduced TrOCs.

## 1. Introduction

In recent years, several organic compounds have been detected in different water bodies—for instance, industrial chemicals, surface treatment agents, pesticides, biocides as well as human and veterinary medicinal products. Since approximately 30% of the drinking water in Germany and even 60% in the federal state North-Rhine-Westphalia is produced from surface water, groundwater recharge and bank filtrate [1], the removal of trace organic contaminants (TrOCs) is often crucial for maintaining drinking water quality. Although below drinking water limits, the presence of TrOCs is of hygienic and esthetical relevance as it is an indicator for a wastewater impact on drinking water and, consequently, discourages consumers from drinking the water.

The water supplier Stadtwerke Düsseldorf AG (SWD) operates three waterworks in the Rhine catchment area, which mainly treat source water containing up to 70% riverbank filtrate for drinking water production. Riverbank filtration is known to eliminate organic contaminants through both biodegradation and sorption on soil. However, some organic micropollutants, e.g., TrOCs, find their way from the Rhine into the source water of the downstream-located drinking water treatment plants (here, referred to as raw water). According to German guidelines [2], organic substances that are not eliminated by natural processes before they reach the waterworks are known as drinking-water-relevant compounds. For instance, they contain PMT (persistent–mobile–toxic) substances, which are environmentally persistent compounds, mobile in the water cycle and toxic to humans [3]. Therefore, the removal of drinking-water-relevant TrOCs before or during drinking water treatment remains a challenge due to their substantially different compositions and properties. Two well-established technologies with versatile and powerful removal capabilities are activated carbon filtration (ACF) and low-pressure reverse osmosis (LPRO).

ACF for drinking water production has been used on an industrial scale in U.S. waterworks since the mid-1920s and in Germany since 1961 [4]. In addition to the removal of non-polar chemicals, odors, off-taste and colorings, the elimination of TrOCs—in particular, pesticides, solvents, drugs and chlorinated hydrocarbons—has attracted attention within the past 15 years [5]. Despite the fact that adsorption is a well-known process, it is quite challenging to predict in detail the breakthrough behavior for single contaminants a priori. This is essentially interpreted by the presence of natural organic matter (NOM), which competes as background substances with TrOCs for the adsorption sites and modifies the surface characteristics of the activated carbon [6]. Accordingly, further studies are needed to obtain information regarding the removal efficiency of different TrOCs at the employed operating conditions in waterworks. Subsequently, certain concerns are considered in this context:

Preferential removal of nonpolar/hydrophobic substances;
Competitive adsorption of TrOCs and background NOM to the activated carbon;
Continuous decrease in activated carbon adsorption capacity with progression of operation time;
Decreasing detention time with increasing filter velocity or reducing empty bed contact time.

LPRO is a pressure-driven membrane process that is increasingly used in water treatment applications, i.e., water softening, desalination and removal of trace organics, in order to produce different water qualities [7,8,9]. The retention of trace organics by LPRO is generally influenced by the physical and chemical properties of the substances, membrane characteristics and operating conditions. In principle, membranes can retain all substances having molecular weights that are higher than the molecular weight cut-off (MWCO) of the membrane [10]. In the case of smaller molecules, physicochemical properties (e.g., charge interactions between membrane and substance, which depend on the degree of dissociation and pH value) determine membrane retention [11,12]. Adsorption processes onto the membrane and/or solubility of small organic substances into the membrane matrix can also adversely affect the rejection [12,13].

Other relevant factors that influence the rejection are the wetting behavior of the membrane, described by the contact angle, the solubility of organic substance in octanol or water, expressed by the logarithmic octanol-water partition coefficient (LOG K_ow_) [14]. Quantitative structure activity relationships (QSAR) is a potential approach developed to predict the rejection of compounds by identifying certain descriptors related to physicochemical properties and through statistical analysis for the experimental results [15,16]. Several attempts have been made in the literature to identify relevant descriptors in order to model and/or predict the rejection of TrOCs [8,17,18,19]. Nevertheless, it is challenging to define the main influencing parameters as they are superimposing [10] and different water qualities should be taken into account [7,9]. ACF loaded with granular activated carbon (GAC) has been part of the drinking water production in waterworks in Düsseldorf for over 50 years. In order to meet future requirements and increasingly stringent regulations regarding drinking water quality, a comparative study was carried out in which the potential of ACF and LPRO technologies for removing of TrOCs was evaluated under comparable conditions using a semi-technical plant. In this work, LPRO and ACF were operated in parallel to avoid complete demineralization of the processed water, and the filtrates of both processes were mixed. The results of a five-month pilot study on the removal of 32 typical TrOCs from Rhine bank filtrates (cf. Table 1) are presented and compared with experiments on a laboratory scale.

## 2. Materials and Methods

### 2.1. Trace Organic Contaminants (TrOCs)

Based on the evaluation of an internal data analysis by SWD, a list of TrOCs relevant to drinking water was created and introduced in Table 1, including basic physicochemical properties.

### 2.2. Membrane

**Figure 1:**
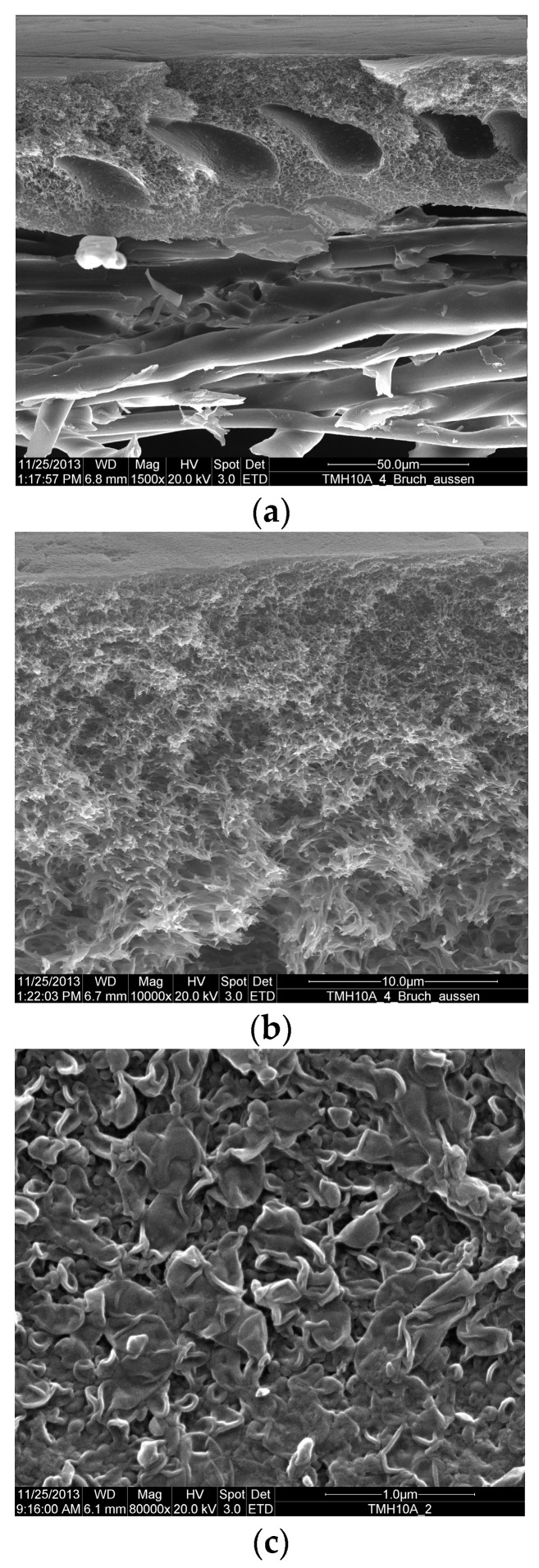
SEM micrographs for the TMH10 membrane: (**a**) overall cross-section for the membrane made of polyamide dense layer, polysulfone support and nonwoven fabric (from top to bottom), (**b**) thin-film composite membrane (polysulfone support and polyamide active layer), (**c**) typical “ridge-and-valley” polyamide surface morphology; measured by University of Duisburg-Essen using a LEICA S420 SEM, Wetzlar, Germany.

Pilot-scale experiments were carried out using 4” LPRO membrane elements, each with 8 m² membrane area (type TMH10H, Toray Membrane Europe AG [21]). Scanning electron microscope (SEM, LEICA S420 SEM, Wetzlar, Germany.) micrographs for a membrane sheet are presented in Figure 1. The membrane comprised a microporous polysulfone base supported by a nonwoven fabric to which a thin, dense film of fully crosslinked aromatic polyamide was applied. The salt rejection as reported by the manufacturer was 99.3%, measured under operating conditions: pressure of 6.9 bar, temperature of 25 °C, feed concentration of 500 mg/L NaCl, recovery rate of 15%, feed water pH 7 and permeate flow rate of 7.2 m^3^/d [21].

### 2.3. Raw Water

The raw water was pumped from five wells; it comprised ~70% Rhine riverbank filtrate and ~30% groundwater. Analysis for the main components in the raw water during experiments was performed and the data are listed in Table 2. Accordingly, the raw water can be considered as hard water (strongly mineralized) with a deviation of 30% in electric conductivity. This was mainly caused by two different wells; one had significant amounts of groundwater with high hardness and total alkalinity, while another had an elevated sodium chloride content due to a second groundwater horizon. In addition, variable iron and manganese concentrations in the raw water were caused by two other wells. This might be critical for the LPRO process, i.e., as a potential source for inorganic fouling, and iron (III) species may deactivate GAC. Therefore, aeration and sand filtration were employed as pretreatment for the removal of iron and manganese species prior to LPRO and ACF.

### 2.4. Pilot Plant

**Figure 2:**
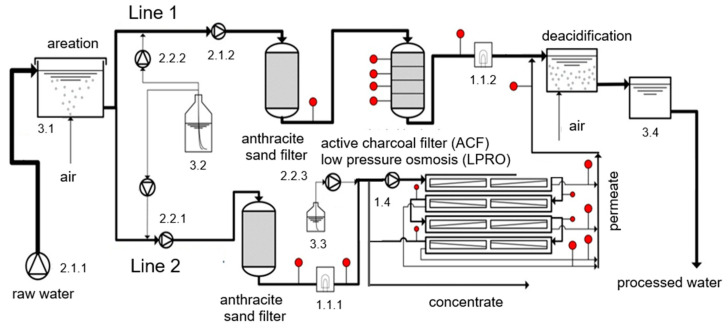
Schematic illustration for the employed pilot plant comprising two treatment lines, ACF (line 1) and LPRO (line 2); red dots refer to sampling points; descriptions of the sampling points are provided in supplementary data (Table S3).

The semi-technical pilot plant was designed by SWD and realized by Cornelsen Umwelttechnologie GmbH, Essen, Germany. The LPRO unit was constructed by Grünbeck Wasseraufbereitung GmbH, Höchstädt a.d. Donau, Germany (GENO-Nano RKF1800 S). The aeration unit was supplied by Aquadosil Wasseraufbereitung GmbH, Essen, Germany. Electrical installation, plant control and data management were installed by SWD. The pilot plant consisted of two treatment lines, ACF and LPRO (cf. Figure 2), with 17 sampling points.

The aerated raw water was continuously pumped from the storage tank (3.1) by the pressure booster pumps 2.1.1 and 2.1.2. TrOCs (3.2) were injected on the suction side of the pumps labeled with 2.2.1 and 2.2.2 to ensure proper flush mixing and good dispersion. The spiked water passed the anthracite sand filters as well as subsequent ACF (line 1) and LPRO (line 2) in parallel mode. The particle filters were backflushed every month. In order to test the effect of UV disinfection as an additional pretreatment step prior to LPRO to reduce biofouling, the UV unit (1.1.1) was operated downstream of the anthracite sand filter. The feed of the LPRO line was pretreated via two cartridge filters consecutively installed with 80 and 5 μm nominal cutoff.

Antiscalant (3.3), DTPMP (phosphonic acid-based), was dosed into the LPRO feed. In large-scale LPRO systems, multiple stages are usually installed, while in pilot-scale, comparable concentration profiles are realized by recirculating the concentrate [9]. The recycling of the concentrate simulates the last membrane elements in a large-scale LPRO plant, where feed concentrations are significantly increased. The specifications of the materials used in the pilot plant are summarized in Table 3.

The removal of TrOCs using ACF is influenced by various operating parameters, e.g., specific throughput and empty bed contact time, which are controlled by the flow velocity. Since the employed empty bed flow velocity was constant at 10 m/h throughout the test period, the specific throughput of ACF and consequently the removal efficiency based on three scenarios, namely low, medium and high specific throughput, were taken into account. For logistic reasons, the test periods with GAC were carried out at 13.9–17.2 m³/kg and 15.2–17.9 m³/kg. The GAC was then renewed and two further experiments with lower specific loads were carried out. It is worth mentioning that a maximum of 18 m³/kg treated water per kg of GAC is considered as short operating time for an ACF in a real drinking water treatment plant.

The operation of LPRO started with a recovery of 80%, which was then increased to 83% for long-term operation including the trial period. The fluxes were adjusted to 25 and 31 L/m²·h, yielding permeate flows of 1.6 and 2.0 m³/h and feed pressures of 8.5 and 10.7 bar, respectively. For online monitoring of organic fouling and scaling, the net pressure and conductivity in the permeate were measured. The net pressure describes the transmembrane pressure after considering the osmotic pressure. Membrane fouling leads to a high net pressure to maintain the given flux ranges. In addition, the increase in conductivity in the total permeate, especially in the permeate of the last pressure tube, is usually an indicator for scaling; however, changes in raw water quality should be also considered. During the investigation period, the plant was operated without CIP (cleaning-in-place). The salt passage of a LPRO membrane was normalized to consider flux and temperature effects [23]. The required salt transport temperature correction factors were provided by the membrane manufacturer.

Spiking experiments using TrOCs were carried out for 5 days to achieve saturation in terms of adsorption for all the employed materials used in the pilot plant, including membranes. In order to avoid/minimize interactions between different substances, TrOCs were classified into three groups, which were dosed sequentially at the spiking concentrations listed in Table 4. Both feed and permeate concentrations were analyzed and the removal of TrOCs calculated. With regard to the removal calculations of TrOCs with concentrations below the limit of quantification (LOQ), numerical values in the permeates were set to LOQ. Accordingly, it is assumed that the resulting rejection rates are higher than the worst case and marked with “>”. 

In addition, the ACF filtrate and the LPRO permeate were mixed, de-acidified by crossflow aeration and stored in a tank (3.4, Figure 2) for further investigation.

### 2.5. Preparation of Stock Solutions

All substances were of analytical reagent grade and were purchased as depicted in supplementary data (Table S1). Perfluorinated compounds (PFAS) were dissolved in 10 mL of dimethylformamide (DMF), ACS reagent, (Sigma-Aldrich, St. Louis, MO, USA).

Then, 2 mL of the stock solution was dissolved in 20 L of deionized water. The solution was then continuously dosed into aerated raw water of lines 1 and 2. For nitrosamines, volatiles and solid substances, methanol, for HPLC, ≥99.9% was used as solvent, whereas aminopolycarboxylic acids were dissolved directly in deionized water. In order to minimize the loss of volatile substances, the stock solutions were stored at 8 °C and replaced after three days. The dosage of TrOCs in the respective experiment phase was carried out using a calibrated cassette hose pump and using Tygon tubes at 60–100 mL/h.

### 2.6. Membrane Autopsy

Membrane autopsies were collected from the leading and last membrane elements at a specific throughput (cumulative permeate volume divided by membrane surface area) of 92 m³/m². They were opened lengthwise; the membrane module was removed, unrolled and samples of 4 and 100 cm² were cut in the front and end spots of the membrane under sterile conditions [19]. The membrane samples were analyzed using SEM-EDX, ATP (adenosine triphosphate), TOC and ICP-OES. For ATP analysis, a measure for actively growing microorganisms, a membrane sample (4 cm²) was sonicated three times for 15 min in 13 mL of 0.9% brine solution. ATP (pg/cm²) in the eluate was determined using ATP Bioluminescence Assay Kits (HS II, Roche Applied Science, Indianapolis, USA). For the TOC analysis, a measure for the organic loading/fouling, a membrane sample (100 cm²) was sonicated for 30 min in 50 mL of deionized water and then diluted 1:10. The eluate was then filtered through a 0.45 µm disc and analyzed according to DIN EN 1484-1997-08 [24]. For the ICP-OES analysis according to DIN EN ISO 11885-2009-09 [25], q membrane samples (100 cm²) were sonicated for 15 min in 50 mL of 10% nitric acid, and after 3 days, the eluate was diluted to 100 mL and filtered. The measurement uncertainty for the analytical results is listed in supplementary data (Table S4).

## 3. Results and Discussion

### 3.1. Performance Measurements during LPRO Operation

**Figure 3:**
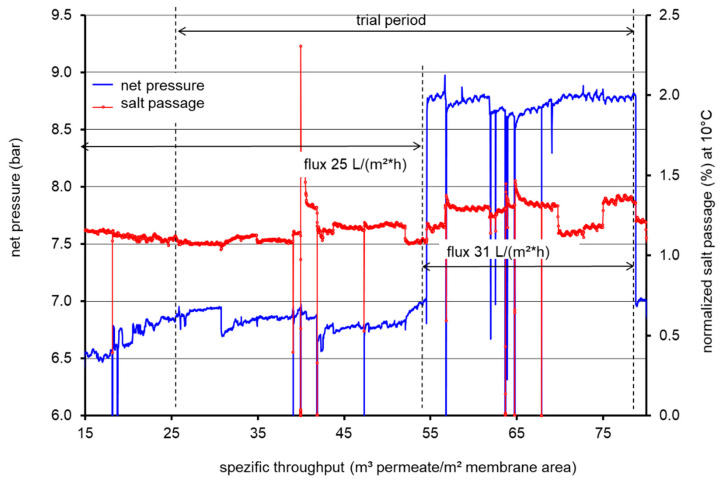
Net pressure and normalized salt passage for LPRO operation during the piloting period of 5 months; zero pressure indicates system shutdowns.

The net pressure and normalized salt passage for LPRO operation during the piloting period of 5 months were plotted vs. specific throughput; curves are presented in Figure 3. A specific throughput of 15 m³ permeate /m² membrane is equivalent to an operation period of one month.

The net pressure values showed limited increase/changes during the LPRO operation for the two fluxes used. The first examination period, characterized by a mean flux of 25 L/m² h, was indicated by a specific throughput from 15 to 55 m³/m² which corresponds to a runtime of around 70 days. During this time, the feed pressure increased slightly from 6.5 to 7.0 bar, which was probably caused by fouling effects. After a specific throughput of 55 m³/m², the feed pressure was increased to 8.8 bar, which triggered an increase in the mean flux to 31 L/m² h. Brief disruptions in net pressure and salt rejection were caused by system shutdowns, while minor peaks resulted from switching the pumping stations. If raw water wells with higher electrical conductivity are used, the conductivity in the feed and in the permeate will decrease. The net pressure behavior indicates the limited fouling or scaling during the piloting period; more insights will be revealed by the membrane autopsy (cf. Section 3.2). Furthermore, the salt passage was observed to be nearly stable during LPRO at the employed fluxes. Nevertheless, a limited increase in the average salt passage at higher flux might be attributed to the increased applied pressure and/or a possible tendency for a limited concentration polarization.

Moreover, the LPRO operation produced permeates having generally an electric conductivity in the range of 16–18 µS/cm (at 25 °C). Exemplary analysis results of the permeate water for the two employed fluxes used are presented in Table 5.

The rejection of divalent ions (calcium, magnesium and sulfate) and silicates increased with flux, while a rather stable rejection behavior was observed for the monovalent ions (sodium, nitrate and chloride). On the other hand, the measured boron rejection was lower than the reported boron rejection (36/59%) for ESPA2/ESPAB LPRO membranes from Hydranautics/Nitto (Prague, Czech Republic) [26]. Nonionized boric acid is supposed to be the dominant species at pH range of 7–8. According to Fuijoka et al., the rejection of neutral and uncharged solutes is influenced by free-volume hole size, free-volume fraction and the active layer thickness [27]. As a result, different rejection ratios for boron could be more related to the intrinsic properties of the various polyamide active layers.

### 3.2. Membranes Autopsy

A visual examination of the employed membrane modules used revealed slimy pale brown deposits on flat membrane sheets, on the upstream side. The autopsies were collected from the leading and the last membrane modules for analysis as described in Section 2.6. The results of elemental, TOC and ATP analysis are shown in Table 6. Higher loadings of fouling substances were identified by ATP and TOC. In addition, aluminum and iron were observed in the leading module. Higher ATP indicates higher biofouling in the leading module than the last module, as expected and always reported in the literature [28,29]. In contrast, higher loadings/deposits of scaling substances, calcium, silicate, barium and strontium were found in the last element compared to the leading module, which implies higher scaling and inorganic fouling in the last module because of elevated cation concentrations upstream of the last module and exceeding the K_sp_ (solubility product). Analysis of membrane samples using SEM and EDX revealed comparable outputs (see supplementary data, Figure S1). Moreover, more phosphate deposits and some DTPMP-Ca-deposits were measured at the last module compared to the leading module. Vrouwenvelder et al. [23] investigated the fouling of different 4” membrane elements in a test rig operated with pre-treated ground water as feed and found approximately 790 to 8900 pg ATP/cm^2^ after 16 to 55 days’ running time, which is in the range of the results presented here. From data analysis, they concluded that a net pressure increase of >10% is expected above 1000 pg/cm² ATP.

### 3.3. Removal Efficiency of TrOCs

#### 3.3.1. Rejection of TrOCs via LPRO Operation

The removal values of TrOCs during LPRO operation are summarized in Table 7 (respective values for ACF processes are discussed in the next section). In most cases, permeate and retentate concentrations for the different pressure vessels were determined (see supplementary data, Table S5), and subsequently, the specific removal values of TrOCs for different vessels were calculated.

It was found that the rejection of group 1, 2 and 3 of TrOCs was not influenced by the variation in the raw water quality, as seen in the cases of BTA (62–65%), DMS (95–97%) and DTPA (87–92%); variations were within the measurement uncertainty (see Supplementary Data, Table S4). In addition, higher apparent rejection rates for TrOCs were not measured when the flux was increased from 25 to 31 L/m²·h.

**Figure 4:**
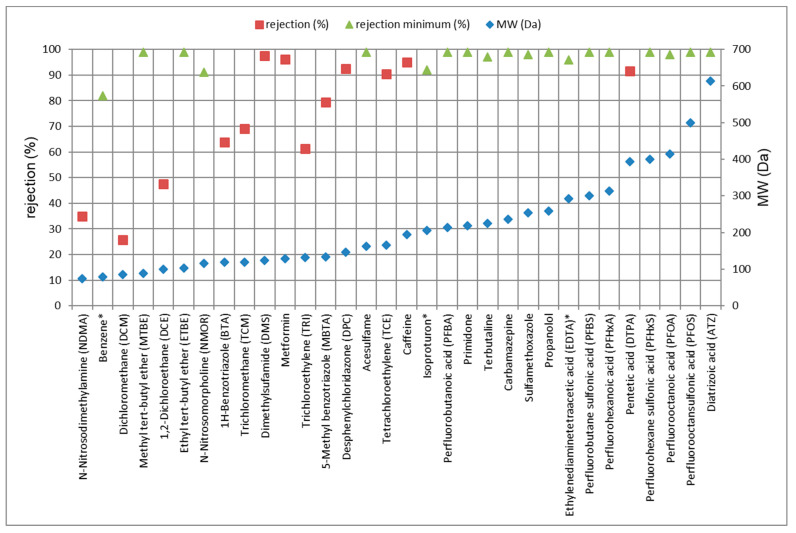
Relationship between molecular weight of TrOCs and rejection values measured at 31 L/m²·h, with the exception of those with sign (*) measured at 25 L/m²·h. The minimum rejection refers to the calculated values using LOQ.

One of the major parameters influencing the retention of TrOCs via LPRO is the relation of the TrOCs molecular weight and MWCO of the RO membrane [10]. For the employed LPRO membranes, MWCO is reported to be approximately 200 g/mol (Da) [30]. Figure 4 presents the relation between molecular weight values of TrOCs and the measured rejection values. It was found that TrOCs exhibiting molecular weight ≥ 150 Da were completely retained, except DTPA (90%). Such superior rejection performance is mainly interpreted in terms of the size exclusion mechanism, which prevailed over any possible impact of surface charges and hydrophobicity [17]. Concerning NMOR, MTBE, ETBE, caffeine, isoproturon, terbutaline, carbamazepine and sulfamethoxazole, the rejection rates are in good accordance with the work by Kegel et al., employing Trisep ACM5 membranes [31]. Nevertheless, better rejection values were measured for NDMA (74%) and benzene (88%), compared to [31], since the LPRO membrane employed here was tighter.

Furthermore, TrOCs exhibiting lower molecular weight values than RO membranes’ MWCO, i.e., DCM, DCE, TCM and TRI, were not properly retained. Samadi et al. measured rejection rates between 64% and 88% for TCM employing a Perma-Pure membrane (MWCO 300 Da) [32]. Nevertheless, a direct correlation between the molecular weight of chlorinated compounds and the measured rejection rates was observed; the rejection rates for DCM < DCE < TCM < TRI increased with increasing molecular weight (cf. Figure 4).

Concerning BTA and TCM, although they have different structures and physical properties, both substances showed comparable rejection rates of 63% and 67%, respectively. This proves the dominant influence of molecular weight. The rejection ratio for MBTA was 15% better than for BTA, which is related to the difference in molecular weight by 14 g/mol. This is in agreement with a study in which BTA was retained by 66% employing a UF/RO system [33].

The polycarboxylic acid DTPA (393 Da) was retained by 87–89%, while EDTA (292 Da), a structurally comparable compound, was retained >7% more. Both substances are bivalent anions according to their pK_a_ values and can form complexes with calcium ions in the feed water. The different rejection ratios may be related to the complexation constants (EDTA/DTPA). The rejection rate for EDTA is in accordance with a study by Müller et al. in a pilot-scale treatment plant [34].

Moreover, the unexpected very different retention rates measured for DCM and MTBE as well as DCE and ETBE, despite their small molecular weight difference, may imply the contribution of other parameters in the retention mechanism, i.e., size exclusion is not the only mechanism here. Klüpfel related the different rejection rates measured for TrOCs, having similar molecular weight values, to their LOG K_ow_ values [35]; higher LOG K_ow_ values resulted in lower rejection rates. The data of the current study support, to a large extent, this hypothesis. Nevertheless, the low rejection ratios of BTA in relation to MTBE may not be explained on this basis. In addition, the rejection ratio for DMS was higher than MBTA (by 13%), although MBTA has a higher molecular weight than DMS that may be explained by the higher polarity of DMS as depicted by the LOG K_ow_ values. Likewise, the different rejection ratios for MBTA and metformin could be also correlated to their LOG K_ow_ values. An analogous concept might also apply to benzene, which was retained by ~82% [36].

According to Xu et al. [18], the rejection rates for TCM and TRI decreased dramatically (from > 80% to < 5%) after 24 h of operation when the dosing concentrations were 80 µg/L; this was attributed to strong solute–membrane interactions. Here, the first sampling was made after 5 days; however, the results did not reveal the same phenomenon. Instead, it was found that the rejection rates for di- and trichloro compounds, i.e., DCM, DCE, TCM and TRI at a 5 µg/L-level, were found to be improved upon increasing the operation time, e.g., in the case of DCM of 23%. This might be related to the formation of a combined fouling layer, comprising organic and inorganic substances, time or filtration progression, a phenomenon called cake-reduced concentration polarization [37].

In addition, low rejection values were measured for NDMA (74 Da): 21% at 25 L/m²·h and 35% at 31 L/m²·h. Rejection ratios for NDMA in the literature, by ESPA2, LFC3, TFC-HR, 70LW and NF-90 membranes, were reported to be in the range of 37% to 52% [7,38]. Takeuchi et al. reported variable removal of NDMA from 20% to 88% by the RO processes [39]. Fujioka et al. studied and compared the rejection of NDMA and boron (as boric acid at pH 6–8) [27,38,40]. These small and neutral molecules (74 and 62 Da, respectively) were found to be very hydrophilic [27], and the size exclusion mechanism (based on free-volume hole-size) was considered to be the main retention mechanism [26,40]. Therefore, comparable rejection ratios of NDMA and boron were found for different commercial RO and NF membranes [27]. Similarly, the same behavior could be, in principle, confirmed in the current study; see Table 5 and Table 7. Nevertheless, the influence of increasing the flux was relatively not the same; the rejection of NDMA was increased from 21% to 35%, while the rejection of boron was decreased from 30.6% to 27.7%.

#### 3.3.2. Removal of TrOCs via ACF Process

The removal values of TrOCs during the ACF process are introduced in Table 7, the data being sorted according to the increase in specific loading of ACF. In general, ACF showed significant removal performance for most TrOCs, including substances that were not properly retained by LPRO, e.g., DCM, DCE, BTA and TCM. On the other hand, ACF was not able to remove salts as expected, which is reflected in the comparable electric conductivities of raw water and processed/treated water. Nevertheless, in general, ACF operation is less energy demanding than LPRO; the pressure drop of ACF was below <1 bar.

Removal of TrOCs by ACF was emphasized to depend on the specific throughput (Table 7). The highest removal was always measured at the beginning of the process for a filter filled with virgin GAC; then, it decreased during the operation in some cases. This effect was only obvious for DMS, metformin, PFBA, DCM, NDMA and EDTA because of the limited operation time in this study. Whereas excellent removal of all other TrOCs by ACF was found, removal of NMOR, MTBE, ETBE, caffeine, isoproturon, terbutaline, carbamazepine and sulfamethoxazole was in good accordance with Kegel et al. [31], where Norit Row Supra 0.8 carbon was employed. Nevertheless, higher removal of NDMA was measured here. Metformin, EDTA and DMS are rather polar substances (i.e., LOG K_ow_ < −1), and consequently, the breakthrough occurred already at specific load of 8.1 m³/kg.

DCM having a LOG K_OW_ of 1.96 was one of two TrOCs whose removal values varied by changing the GAC filling. By increasing the operation time of ACF, a reduction in the removal from 81% to 50% (2nd filling) and 70% to 93% (1st filling) was observed. As a consequence, the differences might be related to the differences between the two AC charges, as well as to the errors of sampling and analytics, which are documented to some extent by the double measurement of the first filling. For metformin, a non-systematic variation of removal from 63% to 97% with specific loading was found.

Moreover, it was found that the risk of breakthrough for TrOCs, especially those with high polarity, was increased for ACFs with high specific throughputs. In the case of riverbank filtrates, this may also occur due to accidental pollution of the corresponding river.

**Figure 5:**
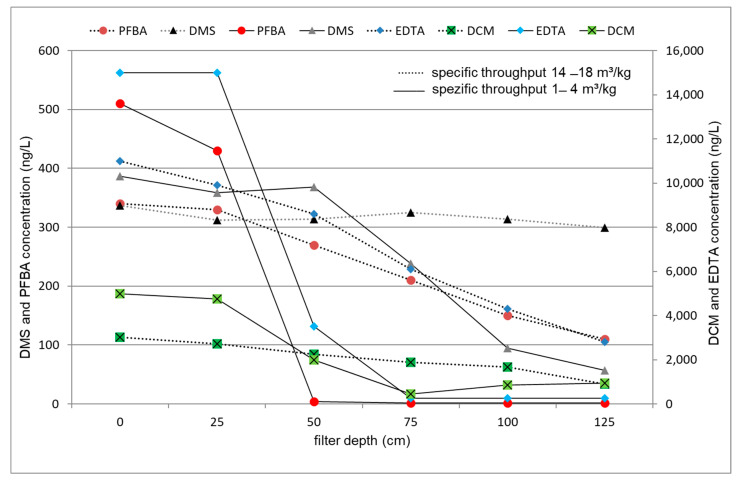
Depth-dependent concentration profiles for TrOCs in ACF filter.

Using virgin GAC, decreasing concentration profiles (i.e., increasing removal trends) were observed for most of the TrOCs along the filter depth (cf. Figure 5). It was also found that the mass transfer zone (MTZ) is sharp for PFBA and EDTA but wide for DMS and DCM; see Section 3.4.2 for further information. At a specific throughput of 14–18 m³/kg, the upper layer was saturated by PFBA and EDTA occurred. DMS, a low-molecular and polar compound with LOG K_ow_ of −1.16, was no more retained by ACF. In contrast, DCM, also a small and polar compound with a LOG K_ow_ of 1.5, was completely adsorbed, as shown in Figure 5.

### 3.4. Evaluation of the Internal Plant Concentration Profiles

#### 3.4.1. LPRO Operation

**Figure 6:**
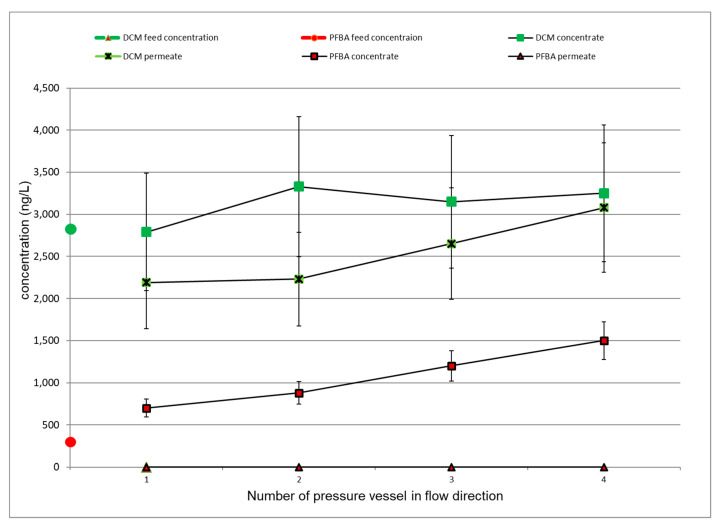
Internal plant concentration profiles for perfluorobutane carboxylic acid (PFBA) and dichloromethane (DCM) with estimated errors.

Along the pressure vessels of the LPRO unit, TrOCs were concentrated in the flow direction on the feed side of the membrane. This is less important for TrOCs with low rejection. This phenomenon is exemplified in Figure 6 by PFBS (a substance with a high rejection) and DCM (a substance with low rejection). The diagram shows the concentration profiles of both substances in feed, permeate and bulk/concentrate of the four pressure vessels. PFBA concentration was increased five times in the concentrate of the last pressure vessel, while DCM concentration was only slightly enhanced.

Moreover, PFBA concentration in the permeates of respective pressure vessels was always below the detection limit (i.e., high rejection), whereas DCM concentration, which started at a high level, increased in the flow direction and even exceeded the feed concentration in the effluent of the last pressure vessel, potentially due to cake-enhanced concentration polarization effects. This result reveals the technical limitation of LPRO operation with respect to the rejection of low-molecular-weight halogenated TrOCs [11]. In the case of higher permeate yields, those poorly removable substances are found in the permeate in even higher concentrations. Higher rejections might be possible by employing a permeate staged system [30], which, in fact, is not applied in many public water treatment plants because of the additional energy consumption.

**Figure 7:**
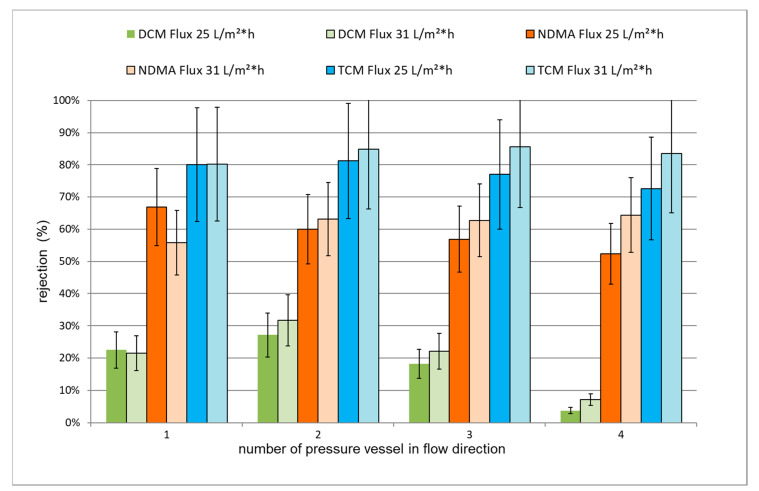
Apparent rejection for exemplary TrOCs at different fluxes for different pressure vessels with estimated errors.

In addition, the rejection of DCM and NDMA by each pressure vessel was measured individually at different mean fluxes, 25 L/m²·h and 31 L/m²·h. The results are shown in Figure 7. By increasing the mean flux, an improvement (up to 12%) in the rejection of DCM and NDMA by pressure vessels 2 to 4 was observed. It is believed that this is related to the contribution of membrane fouling, as it was found that the rejection is independent of the flux in the case of the first pressure vessel, which by design had the highest flow. Therefore, the rejection of TrOCs is strongly influenced by the operating conditions and the membrane state.

#### 3.4.2. ACF

For the consideration of TrOC removal in ACF, depth-dependent concentration profiles are presented in Figure 5. EDTA and PFBA had a sharp adsorption zone (i.e., sharp mass transfer zone, MTZ, cf. Section 3.3.2) with a specific throughput of 1–4 m³/kg, while broad adsorption profiles were observed for DMS and DCM. In principle, sharp MTZ are usually desirable because ACF operation time can be prolonged, without reaching the breakthrough [5]. This is evident in the case of specific throughput 14–18 m³/kg, where an almost complete breakthrough for DMS and a 50% breakthrough for DCM were found. Such time-dependent behavior is considered as a disadvantage for the ACF treatment. This could be overcome by employing two ACFs in series, which is not common in drinking water treatment plants in Germany. In the case of intense loading of well-adsorbable substances at high specific throughputs, the release of weakly adsorbed substances may occur, resulting in a contamination of the drinking water with TrOCs.

Additionally, a further assessment of the data collected at the pilot plant revealed that certain poorly removable TrOCs using ACF were indeed well-retained by the LPRO unit and vice versa. For example, only ACF effectively eliminated DCM, while LPRO is the preferred method for DMS (Table 7). Remineralization can then be maintained by mixing LPRO permeate and the ACF-treated bypass to produce drinking water with adjustable hardness and reduced TrOCs that meet common drinking water guidelines. Accordingly, reverse osmosis and activated carbon can synergistically improve the removal of TrOCs and possibly improve the drinking water [31].

## 4. Summary and Conclusions

The performance of low-pressure reverse osmosis (LPRO) and active carbon filtration (ACF) processes during five-month pilot filtration of Rhine bank filtrates spiked with 32 typical trace organic contaminants (TrOCs) was comprehensively studied. LPRO exhibited nearly stable net pressure behavior with limited membrane fouling. LPRO showed improved rejection for certain divalent ions and silicates with increasing flux, while the rejection of monovalent ions was rather constant. Membrane autopsy revealed higher biofouling in the leading module, whereas higher scaling deposits of calcium, silicate, barium and strontium were detected in the last module. The rejection of TrOCs by LPRO was properly related to TrOCs molar masses and LOG K_ow_ values. Nonetheless, the low rejection rates for dichloro and trichloro compounds by LPRO should be properly considered because volatile halogenated compounds are widely detected in groundwater. On the other hand, ACF showed significant removal performance for most of the TrOCs, including those that were not properly retained by LPRO, while salts could not be removed. The removal of TrOCs by ACF was emphasized to depend on the specific throughput; the highest TrOC removal was always measured at the beginning of the process. Accordingly, advantages and drawbacks for each process in relation to its application in drinking water production are listed in Table 8.

Depending on the site-specific demand, a parallel operation of ACF and LPRO can potentially be an optimal solution. In this case, the disadvantages of one method are partially offset by the advantages of the other—for example, the necessary salting of the permeate by the ACF substream, which leads to a mixed water with medium hardness. This is of particular interest when both TrOCs and hardness are to be reduced.

## Figures and Tables

**Table 1:** List of TrOCs investigated in this work and their physicochemical properties.

Compound ^1^	Molecular Weight (g/mol)	LOG K_OW_^2^	pKa ^3^	Charge at pH 7 ^4^
Group 1				
Perfluorobutanoic acid (PFBA)	214.05	2.25	0.08–0.4	a
Perfluorohexane sulfonic acid (PFHxS)	400.14	3.68	0.14	a
Perfluorooctanoic acid (PFOA)	414.09	4.93	1.3	a
Perfluorobutane sulfonic acid (PFBS)	300.12	2.34	−3.13	a
Perfluorohexanoic acid (PFHxA)	314.07	3.59	−0.16	a
Perfluorooctansulfonic acid (PFOS)	500.16	5.02	0.14	a
Group 2				
1H-Benzotriazole (BTA)	119.14	1.02	8.20	n
Dimethylsufamide (DMS)	124.19	−1.16	10.4	n
5-Methyl benzotriazole (MBTA)	133.15	1.4	8.66	n
Metformin	129.16	−1.27	12.4	c
Desphenylchloridazone (DPC)	145.56	−0.35	9.29	n
Acesulfame	163.17	−0.34	2.0	a
Caffeine	194.19	−0.10	10.4	n
Isoproturon	206.32	2.87	15.06	n
Primidone	218.28	0.91	12.3	n
Terbutaline	225.32	0.9	8.80	c
Carbamazepine	236.29	2.45	13.94	n
Sulfamethoxazole	253.31	0.89	5.60	n
Propanolol	259.34	2.98	9.46	c
Diatrizoic acid (ATZ)	613.93	1.75	0.92	a
Group 3				
N-Nitrosodimethylamine (NDMA)	74.1	−0.57	3.22	n
Benzene	78.12	2.13	-	n
Dichloromethane (DCM)	84.94	1.5	-	n
Methyl tert-butyl ether (MTBE)	88.17	0.94	-	n
1,2-Dichloroethane (DCE)	98.97	1.48	-	n
Ethyl tert-butyl ether (ETBE)	102.2	1.43	-	n
N-Nitrosomorpholine (NMOR)	116.14	−0.44	3.14	n
Trichloromethane (TCM)	119.38	1.97	15.5	n
Trichloroethylene (TRI)	131.39	2.61	25–27	n
Tetrachloroethylene (TCE)	165.83	3.40	14	n
Ethylenediaminetetraacetic acid (EDTA)	292.28	−5.88	2.0; 2.7; 6.16; 10.26	a
Pentetic acid (DTPA)	393.4	−8.53	~1.80	a

^1^ Suppliers for TrOC substances are provided in supplementary data (Table S1); ^2^ Calculated using Virtual Computational Chemistry Laboratory according to the literature [20]; ^3^ References for pKa values are available in supplementary data (Table S2); ^4^ Charge in water at pH 7, a: anionic, c: cationic, n: neutral.

**Table 2:** Chemical composition and main properties of the raw water.

Parameter ^1^	Number of Analyses	Minimum	Maximum	Mean	Median
Temperature (°C)	63	10.7	13.7	12.0	11.9
pH	32	7.12	7.49	7.31	7.30
Oxygen (mg/L)	11	3.0	3.9	2.7	2.8
Electric conductivity (µS/cm)	55	619	920	803	803
Total acidity (mM)	16	0.41	0.81	0.62	0.63
Total alkalinity (mg/L CaCO_3_)	13	183	226	203	201
Calcium (mg/L)	14	83.0	108	96.6	97.0
Magnesium (mg/L)	14	11.6	14.3	13.2	13.4
Sodium (mg/L)	14	36.7	61.2	48.4	48.7
Potassium	14	4.12	4.89	4.39	4.33
Ammonia (mg/L)	6	0.010	0.030	0.015	0.010
Iron (mg/L)	14	0.0075	0.11	0.015	0.0075
Manganese (mg/L)	14	0.0015	0.11	0.016	0.0015
Nitrate (mg/L)	25	14.9	33.3	21.8	21.5
Silicon dioxide (mg/L)	14	8.1	11.0	10.0	10.2
Sulfate (mg/L)	25	44.0	71.0	61.5	61.0
Chloride (mg/L)	24	69	121	92	89
Fluoride (mg/L)	25	0.11	0.17	0.15	0.15
Bromide (mg/L)	3	0.11	0.14	0.13	0.14
Phosphate total (mg/L)	12	0.044	0.060	0.051	0.050
Total organic carbon (mg/L)	5	0.55	0.79	0.64	0.64
Total plate count at 20 °C (cfu/mL)	63	0	6	0.33	0
Total plate count at 36 °C (cfu/mL)	63	0	3	0.14	0

^1^ Methods employed were according to the German standard methods for the examination of water, wastewater and sludge [22].

**Table 3:** Specifications of the materials used in the pilot plant.

Treatment Step	Material
Anthracite sand filter (line 1/2)	10 cm gravel, 30 mm and 80 cm silica sand, 0.71–1.25 mm; Euroquarz, Dorsten, Germany
	70 cm Hydroanthrazit; 1.4–2.5 mm (Euroquarz)
GAC filter	750 L Filtrasorb F-400, Chemviron Carbon GmbH, Beverungen, Germany, specific surface:1050–1200 m²/g, bulk density: 0.425 g/cm³
LPRO	Eight 4-inch elements TMH 10A (Toray Membrane Europe AG [21]) of 8 m² each in four pressure vessels.
Antiscalant	Ropur RPI 2000A, Münchenstein, Switzerland and MT 4000, Grünbeck, Höchstädt a.d. Donau, Germany (active substance: phosphonic acid, DTPMP) with 0.2 g P/m³ in the feed

**Table 4:** Classification of TrOCs, spiking concentrations and analysis methods.

Group	Substance Class	Number of Substances	Spiking Concentration (µg/L)	Method of Analysis^3^
1	Polyfluorinated aliphatic compounds ^1^	6	0.5	HPLC/MS after SPE
2	Pharmaceuticals, pesticides and metabolites ^2^	14	1	UPLC-MS/MS, direct injection
3	Volatile organic compounds (VOC) Nitrosamines Aminopolycarboxylic acids	8 2 2	5 0.02 10	SPME-GC/MS GC/MS after SPE GC/PND after liquid–liquid extraction

^1^ PFOA, PFOS: 0.1 µg/L; ^2^ carbamazepine, isoproturon: 0.1 µg/L; ^3^ measurement uncertainty data are available in supplementary data (Table S4).

**Table 5:** Inorganic parameter of filter drains, ACF drain and LPRO permeates at two fluxes of 25 and 31 L/m²·h s.

Parameter ^1^	Effluent Anthracite Sand Filter 1	Permeate	Reduction by LPRO (%)	Effluent Anthracite Sand Filter 1	Permeate	Reduction by LPRO (%)
	25 L/m²·h			31 L/m²·h		
Electric conductivity (µS/cm)	713	16.7	97.7	704	14.6	97.9
Calcium (mg/L)	95	1.0	98.9	87	<1	>98.8
Magnesium (mg/L)	13.1	0.1	99.2	12.3	<0.1	>99.1
Sodium (mg/L)	45.9	2.4	94.8	35.5	1.80	94.9
Potassium (mg/L)	4.9	0.21	95.7	4.2	0.20	95.3
Nitrate (mg/L)	14.7	2.4	83.7	14.9	2.5	83.2
Silicon dioxide (mg/L)	13.29	0.43	96.8	11.6	0.18	98.4
Sulfate (mg/L)	55	3.9	92.9	55	1.0	99.1
Chloride (mg/L)	101	3.7	96.3	77	3.0	96.1
Fluoride (mg/L)	0.14	<0.1	>28.6	0.16	<0.1	>37.5
Boron (mg/L)	0.049	0.034	30.6	0.047	0.034	27.7

^1^ The measurements are carried out according to the German standard methods for investigating water, wastewater and sludge, Beuth Verlag, Berlin, Germany.

**Table 6:** Detection of fouling and scaling relevant parameters on autopsied membranes.

Element Autopsy Position	Leading Module			Last Module		
	(Front)	(Mid)	(End)	(Front)	(Mid)	(End)
Calcium (µg/cm²)	4.0	5.0	4.0	7.0	12	14
Phosphorus total (µg/cm² PO_4_)	3.0	3.7	2.6	6.8	12	15
Silicon (µg/cm² SiO_2_)	0.52	0.59	0.70	0.79	0.88	0.91
Aluminum (µg/cm²)	0.34	0.41	0.39	0.32	0.32	0.28
Iron (µg/cm²)	0.36	0.38	0.40	0.29	0.35	0.33
Strontium (ng/cm²)	60	80	98	90	119	103
Barium (ng/cm²)	30	37	32	39	53	57
TOC (µg/cm²)	33	41	30	29	27	27
ATP (pg/cm²)	560	550	500	250	280	300

**Table 7:** Removal values (%) of TrOCs by LPRO (for different fluxes) and ACF processes (ACF data sorted according to the increase in the specific load of ACF).

**Specific loading for perfluoro compounds (m³/kg)**				1.1	6.2	13.9
**Specific loading for poly carboxylic acids and nitrosamines (m³/kg)**				3.7	9.4	17.9
**Specific loading for solid compounds (m³/kg)**				2.4	8.1	14.8–15.2
**Specific loading for volatiles (m³/kg)**				3.7	9.4	17.2–17.9
**Flux (L/m² h)**	25	25	31			
**Sequence of experiments**	1	2	3	2	3	1
**Compound ^1^**	**LPRO**			**ACF**		
*N*-Nitrosodimethylamine (NDMA)	21	-	35	-	60	70
Benzene	82/80	82	-	>99	-	>99/>99
Dichloromethane (DCM)	3.2/4.9	26	26	81	50	93/70
Methyl tert-butyl ether (MTBE)	99/>99	>99	>99	>99	96	92/87
1,2-Dichloroethane (DCE)	29/21	45	47	>99	>99	>99/>99
Ethyl tert-butyl ether (ETBE)	>99/>99	>99	>99	>99	>99	>99/>99
Acesulfame	>99/>99	>99	>99	>99	>99	>99/>99
*N*-Nitrosomorpholine (NMOR)	95	-	>91	-	>96	98
1H-Benzotriazole (BTA)	63/65	62	64	99	>99	>99/>99
Trichloromethane (TCM)	50/46	65	69	>99	>99	>99/>99
Dimethylsufamide (DMS)	>96/>96	95	97	85	15	8 /11
Metformin	90/91	96	96	82	63	97/87
Trichloroethylene (TRI)	52/48	67	61	>99	>99	>99/99
5-Methyl benzotriazole (MBTA)	80/82	82	79	>99	>99	>99/>99
Desphenylchloridazon (DPC)	88/90	91	92	>98	>98	96/97
Tetrachloroethylene (TCE)	97/>96	91	90	>98	>95	>98/>98
Caffeine	>98/>98	>98	95	>99	97	>99/>99
Isoproturon	90/>90	>91	-	>92	-	>92/>91
Perfluorbutanoic acid (PFBA)	>99	>99	>99	>99	>99	68
Primidone	>98/>98	>98	>99	>98	>99	>99/>99
Terbutaline	>99/>99	>98	>97	>97	>99	>99/>99
Carbamazepine	>92/>92	>93	>99	>93	>99	>93/>92
Sulfamethoxazole	>98/>98	>99	>98	>99	>99	>99/>99
Propranolol	85/>98	>99	>99	>99	>99	97/>99
Ethylenediaminetetraacetic acid (EDTA)	95	>96	-	>96	98	75
Perfluorbutane sulfonic acid (PFBS)	>99	>99	>99	>99	>99	>99
Perfluorohexanoic acid (PFHxA)	>99	>99	>99	>99	>99	>99
Pentetic acid (DTPA)	87	89	92	89	97	>99
Perfluorohexane sulfonic acid (PFHxS)	>99	>99	>99	>99	>99	>99
Perfluorooctanoic acid (PFOA)	>96	>97	>98	>98	>99	>97
Perfluorooctansulfonic acid (PFOS)	>95	>97	>99	>97	>99	>96
Diatrizoic acid (ATZ)	>98/>98	>99	>99	>99	>99	>99/>99

^1^ Species in feed water may differ from the dosed substances according to the stability of anions/cations in the raw water and the pH value. For dosed substances, see supporting information (Table S1).

**Table 8:** Advantages and disadvantages for individual LPRO and ACF processes.

	**LPRO Process**	**ACF** **Process**
**Advantages**	Water softening and desalination Removal of polar compounds Online status monitoring Good predictability of rejection Long life service time	Low energy consumption Good removal of VOCs and non-polar TrOCs Flexible setting of the output volume Easy handling
**Drawbacks**	Low VOC removal High energy demand Remineralization or partial flow operation to match drinking water regulations Limited quantity adjustment Complex procedure Use of antiscalants Disposal of the concentrate Regular cleaning required	No desalination Adsorption profile depends on the substance and cannot be fully predicted No consistent quality Fast breakthrough for highly polar compounds Adsorption capacity check during operation is necessary Regular AC change with reactivation

## Data Availability

Supplementary Materials.

## Supplementary Materials

The following are available online at https://www.mdpi.com/2077-0375/11/1/33/s1, Table S1. Suppliers for TrOCs substances, Table S2. References for pKa values, Table S3. Description for sampling points, Table S4. Uncertainty ranges for the analytical measurements, Table S5. Pipe-specific concentrations for permeates and concentrates, Figure S1. Results of membrane autopsy.
