# Supplementary material for: Removal of Trace Organic Contaminants by Parallel Operation of Reverse Osmosis and Granular Activated Carbon for Drinking Water Treatment
Source: Membranes (Basel). 2021 Jan 2;11(1):33. doi: 10.3390/membranes11010033 (PMC7823482; doi:10.3390/membranes11010033)
Supplement: Supplementary file 1 [file membranes-11-00033-s001.pdf]

membranes

MDPI

Supplementary information

Removal of Trace Organic Contaminants by Parallel Operation of Reverse Osmosis and Granular Activated Carbon for Drinking Water Treatment

Norbert Konradt [1,*], Jan Gerrit Kuhlen [2], Hans-Peter Rohns [1], Birgitt Schmitt [1], Uwe Fischer [1], Timo Binder [1], Vera Schumacher [3], Christoph Wagner [1], Stefan Kamphausen [1], Uwe Müller [4,*], Frank Sacher [4], Peter Janknecht [5], Ralph Hobby [6], Ibrahim M.A. ElSherbiny [6] and Stefan Panglisch [6]

1 Department of Waterworks, Stadtwerke Düsseldorf AG, Wiedfeld 50, 40589 Düsseldorf, Germany; hprohns@swd-ag.de (H.-P.R.); bschmitt@swd-ag.de (B.S.); ufischer@swd-ag.de (U.F.); tbinder@swd-ag.de (T.B.); cwagner@swd-ag.de (C.W.); skamphausen@swd-ag.de (S.K.)

2 Viega Technology GmbH & Co. KG, Viegaplatz 1, 57439 Attendorn, Germany; Jan-Gerrit.Kuhlen@viega.de (J.G.K.)

3 Berliner Wasserbetriebe, Motardstraße 35, 13629 Berlin, Germany; vera.schumacher@bwb.de (V.S.)

4 DVGW-Technologiezentrum Wasser, Karlsruher Straße 84, 76139 Karlsruhe, Germany; frank.sacher@tzw.de (F.S.)

5 Enercity Netz GmbH, Auf der Papenburg 18, 30459 Hannover, Germany; peter.janknecht@enercity-netz.de (P.J.)

6 Chair for Mechanical Process Engineering and Water Technology, University of Duisburg-Essen, 47057 Duisburg, Germany; ralph.hobby@uni-due.de (R.H.); ibrahim.elsherbiny@uni-due.de (I.M.A.E.); stefan.panglisch@uni-due.de (S.P.)

* Correspondence: NKonradt@swd-ag.de (N.K.); uwe.mueller@tzw.de (U.M.)

Received: 24 November 2020; Accepted: 14 December 2020; Published: date

Table S1. Suppliers for TrOCs substances.

Substance	Supplier	Product numbers
Acesulfame potassium	Sigma Aldrich	04054-25g
Diatrizoic acid (ATZ)	Sigma Aldrich	D9268-50g
Benzene	J.T. Baker	9256-02
1H-Benzotriazole (BTA)	Sigma Aldrich	B11400-100g
5-Methyl-1H-benzotriazole (MBTA)	Sigma Aldrich	196304-10g
Carbamazepine	Sigma Aldrich	C4024-1g
Trichloromethane (TCM)	Merck	1.024.441.000
Caffeine	Fluka AG	27600-25g
1,2-Dichloroethane (DCE)	Merck	100955
Dichloromethane (DCM)	Merck	822271
Dimethylsufamide (DMS)	Dr. Ehrenstorfer	C12743000
Desphenylchloridazone (DPC), 99,8%	BASF	14456
Titriplex® V, Pentetic acid	Merck	1.084.260.100
Ethylenediaminetetraacetic acid disodium salt dihydrate	Sigma Aldrich	10631-3
Ethyl tert-butyl ether (ETBE)	Acros Organics	403530250
Iopamidol	Medithek Gmbh	R00901398
Isoproturon	Sigma Aldrich	36137-100mg
1,1-Dimethylbiguanide hydrochloride (Metforminhydrochloride)	Sigma Aldrich	D150959-5g
tert-Butyl methyl ether (MTBE)	Merck	1.018.451.000
N-Nitrosodimethylamine (NDMA)	Sigma Aldrich	48552
Nitrosomorpholine (NMOR)	Sigma Aldrich	N7382-1mL

Heptafluorobutyric acid, Perfluorbutanoic acid (PFBA)	Sigma Aldrich	52411-5mL-f
Tetrabutylammonium nonafluorobutane-sulfonate, Perfluorbutane sulfonic acid (PFBS)	Sigma Aldrich	86909-5g
Undecafluorohexanoic acid, Perfluorohexanonic acid (PFHxA)	Sigma Aldrich	29226-5mL
Tridecafluorohexane-1-sulfonic acid potassium salt	Sigma Aldrich	50929-10g-f
Perfluorooctanoic acid,	Sigma Aldrich	171468-5g
Heptadecafluorooctanesulfonic acid tetraethylammonium salt	Sigma Aldrich	365289-5g
Primidone	Sigma Aldrich	p7295-5g
(±)-Propranolol hydrochloride	Sigma Aldrich	P0884-1g
Sulfamethoxazole	Sigma Aldrich	S7507-10g
Terbutaline hemisulfate salt	Sigma Aldrich	T-2528-1g
Tetrachloroethylene (TCE)	Merck	1.009.641.000
Trichloroethylene (TRI)	Fluka	207581 779

Table S2. References for pKa values.

Compound	Source
**Group 1**	
Perfluorobutanoic acid (PFBA)	A. H. Karoyo; L. D. Wilson Nanomaterials **2015,** *5*, 981-1003
Perfluorohexane sulfonic acid (PFHxS)	ATSDR; Draft Toxicological Profile for Perfluoroalkyls. Atlanta, GA: Agency for Toxic Substances and Disease Registry, US Public Health Service (2015). Available from, as of Oct 5, 2015: http://www.atsdr.cdc.gov/toxprofiles/index.asp
Perfluorooctanoic acid (PFOA)	Kutsuna S., Hori H. Atmos Environ **2008**, *42*, 8883-8892
Perfluorobutane sulfonic acid (PFBS)	Royal Soc Chem; ChemSpider. Nonafluorobutanesulfonic acid. (375-73-5). Available from, as of Dec 15, **2015**: http://www.chemspider.com/Search.aspx
Perfluorohexanoic acid (PFHxA)	Zhao L. et al; Chemosphere **2014**, *114*, 51-58
Perfluorooctansulfonic acid (PFOS)	A. H. Karoyo; L. D. Wilson; Nanomaterials **2015,** *5*, 981-1003
**Group 2**	
1H-Benzotriazole	Katritzky, A. R.; Rachwal S.; Hitchings G. J. Tetrahedron **1991**, *47,* 2683–2732
Dimethylsufamide	D. Trogolo; B. K. Mishra; M. B. Heeb; U. von Gunten; J. S. Arey; Environ. Sci. Technol. **2015**, *49*, 4163–4175
5-Methyl benzotriazole	You-Sheng Liu, Guang-Guo Ying, Ali Shareef, Rai S. Kookana, Environ. Chem. 2013, 10, 135–143
Metformin hydrochloride	Jones OAH et al; Water Res **2002**, *26*, 5013-5022
Desphenylchloridazone	https://www.chemicalbook.com/ChemicalProductProperty_DE_CB8289793.htm
Acesulfame	Walters DE; The Sweetner Book. Gale Walters Publishing, ISBN 978-0989109208 (2013) http://www.sweetenerbook.com/acesulfame.html
Caffeine	Bahrami H; Tabrizchi M; Farrokhpour H Chem Phys **2013**, *415*,222–227
Isoproturon	https://www.chemicalbook.com/ProductChemicalPropertiesCB1113929_EN.htm
Primidone	Schaffer M et al; Chemosphere **2012**, *87*, 513-520
Terbutaline	https://www.chemicalbook.com/ChemicalProductProperty_DE_CB4164487.htm

Carbamazepine	https://m.chemicalbook.com/ChemicalProductProperty_DE_cb1143564.htm
Sulfamethoxazole	https://www.chemicalbook.com/chemicalproductproperty_en_cb6473303.htm
Propanolol	ChemIDplus-database of United States National Library of Medicine (NLM)
Diatrizoic acid	https://www.chemicalbook.com/ChemicalProductProperty_DE_CB9258330.htm
**Group 3**	
N-Nitrosodimethylamine	H. Takeuchi; N. Yamashita; N. Nakada; H. Tanaka; Int. J. Environ. Res. Public Health **2018**, *15*, 1960
N-Nitrosomorpholine	H. Takeuchi; N. Yamashita; N. Nakada; H. Tanaka; Int. J. Environ. Res. Public Health **2018**, *15*, 1960
Trichloromethane	https://www.chemicalbook.com/ChemicalProductProperty_EN_cb5413313.htm
Trichloroethylene	M. Gabričević; G. Lente; I. Fábián J. Phys. Chem. A, **2015**, *119*, 12627
Ethylenediaminetetraacetic acid (H_4EDTA)	Raaflaub, J. Methods Biochem. Anal. **1956**, *3*, 301–324
Pentetic acid (H_5DTPA)	Moeller, T.; Thompson, L. C. Journal of Inorganic and Nuclear Chemistry **1962**, *24*, 499

Table S3. Description for sampling points.

Sampling point	Description
CAF1	Drain filter anthracite/sand, line 1
CUV1	Drain UV, line 1, input LPRO
CNP1	Permeate pressure vessel 1
CNP2	Permeate pressure vessel 2
CNP3	Permeate pressure vessel 3
CNP4	Permeate pressure vessel 4
CNP	Collected permeate LPRO
CNF1	concentrate pressure vessel 1
CNF2	concentrate pressure vessel 2
CNF3	concentrate pressure vessel 3
CNF4	concentrate pressure vessel 4
CAF2	Drain filter anthracite/sand, line 2
CAK2/1	ACF, 1. Sampling point from top
CAK2/2	ACF, 2. Sampling point from top
CAK2/3	ACF, 3. Sampling point from top
CAK2/4	ACF, 4. Sampling point from top
CAAK2	Drain ACF, line 2

Table S4. Uncertainty ranges for the analytical measurements.

**Group 1**	**Relative measurement uncertainty (%)**[1]	**Group 2**	**Relative measurement uncertainty (%)**[1]
**Polyfluorinated aliphatic compounds**		**Pharmaceuticals, pesticides and metabolites**	

Perfluorbutanoate (PFBA)	15	1H-Benzotriazole (BTA)	8
Perfluorohexane sulfonate (PFHxS)	15	Dimethylsufamide (DMS)	32
Perfluorooctanoate (PFOA)	15	5-Methyl benzotriazole (MBTA)	10
Perfluorbutane sulfonate (PFBS)	15	Metformin	18
Perfluorohexanoate (PFHxA)	15	Desphenylchloridazone (DPC)	15
Perfluorooctansulfonate (PFOS)	15	Acesulfame	15
		Caffeine	15
		Isoproturon	30
		Primidone	18
		Terbutaline	27
		Carbamazepine	9
		Sulfamethoxazole	18
		Propanolol	18
		Diatrizoic acid (ATZ)	18

**Group 3**	**Relative measurement uncertainty (%)**[1]	**Parameters of autopsied membrane**	**Relative measurement uncertainty (%)**[1]
**Volatiles**		Calcium	2.5
Benzene	18	Phosphate total	5.0
Dichloromethane (DCM)	25	Silicon dioxide	2.5
1,2-Dichloroethane (DCE)	22	Aluminium	2.5
Trichloromethane (TCM)	22	Iron	2.5
Trichloroethylene (TRI)	20	Strontium	2.5
Tetrachloroethylene (TCE)	9	Barium	2.5
Methyl tert-butyl ether (MTBE)	20	TOC	7.5
Ethyl tert-butyl ether (ETBE)	15	ATP	10
**Nitrosamines**			
N-Nitrosodimethylamine (NDMA)	18		
N-Nitrosomorpholine (NMOR)	18		
**Aminopolycarboxylic acids**			
Ethylenediaminetetraacetic acid (EDTA)	10		
Pentetic acid (DTPA)	10		

[1] Relative standard deviation for the method

Table S5. Pipe-specific concentrations for permeates and concentrates.

Group1 Polyfluorinated organic compounds[1]

Date of experiment 12.01.2011

Parameter [ng/l]	LOQ	CUV1	CNP1	CNP2	CNP3	CNP4	CNP	CNF1	CNF2	CNF3	CNF4	CAF2	CAK2/1	CAK2/2	CAK2/3	CAK2/4	CAAK2
Perfluorobutanoate (PFBA)	2	290	n.d.	n.d.	n.d.	n.d.	n.d.	700	880	1200	1500	340	330	270	210	150	110
Perfluorohexanoate (PFHxA)	2	310	n.d.	n.d.	n.d.	n.d.	n.d.	730	910	1200	1500	360	290	130	35	2	n.d.
Perfluorooctanoate (PFOA)	2	61	n.d.	n.d.	n.d.	n.d.	n.d.	150	180	250	330	70	46	10	n.d.	n.d.	n.d.
Perfluorobutansulfonate (PFBS)	2	310	n.d.	n.d.	n.d.	n.d.	n.d.	720	930	1200	1600	350	270	100	17	n.d.	n.d.
Perfluorohexansulfonate (PFHxS)	2	310	n.d.	n.d.	n.d.	n.d.	n.d.	760	970	1300	1700	360	200	33	n.d.	n.d.	n.d.
Perfluoroctansulfonate (PFOS)	2	48	n.d.	n.d.	n.d.	n.d.	n.d.	120	160	210	290	55	30	4	n.d.	n.d.	n.d.

Date of experiment 02.03.2011

Parameter [ng/l]	LOQ	CUV1	CNP1	CNP2	CNP3	CNP4	CNP	CNF1	CNF2	CNF3	CNF4	CAF2	CAK2/1	CAK2/2	CAK2/3	CAK2/4	CAAK2
Perfluorobutanoate (PFBA)	1	510	-	-	-	4	-	-	-	-	3100	510	430	4	2	2	2
Perfluorohexanoate (PFHxA)	1	430	-	-	-	2	-	-	-	-	1600	430	270	n.d.	n.d.	n.d.	n.d.
Perfluorooctanoate (PFOA)	1	79	-	-	-	n.d.	-	-	-	-	380	83	44	n.d.	2	2	2
Perfluorobutansulfonate (PFBS)	1	420	-	-	-	2	-	-	-	-	1700	410	230	n.d.	n.d.	n.d.	n.d.
Perfluorohexansulfonate (PFHxS)	1	270	-	-	-	2	-	-	-	-	1800	460	220	n.d.	n.d.	n.d.	n.d.
Perfluoroctansulfonate (PFOS)	1	84	-	-	-	n.d.	-	-	-	-	420	80	24	n.d.	n.d.	n.d.	n.d.

Date of experiment 30.03.2011

Parameter [ng/l]	LOQ	CUV1	CNP1	CNP2	CNP3	CNP4	CNP	CNF1	CNF2	CNF3	CNF4	CAF2	CAK2/1	CAK2/2	CAK2/3	CAK2/4	CAAK2
Perfluorobutanoate (PFBA)	1	470	-	-	-	1	-	-	-	-	2300	620	620	380	99	6	1
Perfluorohexanoate (PFHxA)	1	470	-	-	-	1	-	-	-	-	2100	660	640	97	n.d.	n.d.	n.d.
Perfluorooctanoate (PFOA)	1	92	-	-	-	n.d.	-	-	-	-	480	130	120	4	18	n.d.	n.d.
Perfluorobutansulfonate (PFBS)	1	460	-	-	-	2	-	-	-	-	2200	580	580	60	n.d.	n.d.	n.d.
Perfluorohexansulfonate (PFHxS)	1	560	-	-	-	n.d.	-	-	-	-	3000	780	750	14	n.d.	n.d.	n.d.
Perfluoroctansulfonate (PFOS)	1	88	-	-	-	2	-	-	-	-	440	110	110	n.d.	n.d.	n.d.	n.d.

[1] n.d. below LOQ (limit of quantification), n.d. was set for calculation to LOQ/2

Group 2 Pharmaceuticals, pesticides and metabolites

Date of experiment 17.01.2011

Parameter [ng/l]	LOQ	CUV1	CNP1	CNP2	CNP3	CNP4	CNP	CNF1	CNF2	CNF3	CNF4	CAF2	CAK2/1	CAK2/2	CAK2/3	CAK2/4	CAAK2
1H-Benzotriazole	10	870	192	258	347	520	326	1525	1940	2505	3332	1070	12	n.d.	n.d.	n.d.	n.d.
Dimethylsufamide (DMS)	10	315	n.d.	n.d.	n.d.	13	n.d.	665	905	1081	1539	324	326	328	320	324	297
Metformin hydrochloride	10	315	n.d.	16	15	49	32	2995	4270	4609	7973	390	1159	511	140	32	11
Methyl-Benzotriazole	10	955	111	144	194	307	187	1905	2385	3207	4315	1110	n.d.	n.d.	n.d.	n.d.	n.d.
Diglyme	10	31	18	21	n.d.	23	n.d.	80	85	110	142	34	56	12	11	n.d.	29
Desphenylchloridazone (DPC)	10	350	27	30	48	78	41	860	1090	1517	1984	371	66	n.d.	n.d.	n.d.	13
Caffeine	10	890	n.d.	n.d.	n.d.	n.d.	n.d.	1945	2535	3036	4366	1035	65	n.d.	n.d.	n.d.	n.d.
Isoproturon	10	104	n.d.	n.d.	n.d.	n.d.	10	215	280	343	510	134	n.d.	n.d.	n.d.	n.d.	n.d.
Primidone	10	1240	n.d.	n.d.	n.d.	n.d.	n.d.	2550	3160	4221	5851	1415	425	n.d.	n.d.	n.d.	n.d.
Terbutaline sulphate	10	830	n.d.	n.d.	n.d.	n.d.	n.d.	1650	2090	2880	3717	1180	26	n.d.	n.d.	n.d.	n.d.
Diuron	10	10	n.d.	n.d.	n.d.	n.d.	n.d.	30	35	51	64	15	n.d.	n.d.	n.d.	n.d.	n.d.
Carbamazepine	10	134	n.d.	n.d.	n.d.	n.d.	n.d.	280	375	480	667	148	14	n.d.	n.d.	n.d.	n.d.
Sulfamethoxazole	10	640	n.d.	n.d.	n.d.	n.d.	n.d.	1240	1660	2041	3066	1190	263	n.d.	n.d.	n.d.	n.d.
Propanolol	10	720	51	14	87	18	111	1620	2050	2727	3982	1235	n.d.	n.d.	24	13	40
Diatrizoic acid	10	750	n.d.	n.d.	n.d.	n.d.	n.d.	1645	2020	2576	3782	1165	1076	576	200	29	n.d.
Iopamidole	10	36	n.d.	n.d.	n.d.	n.d.	n.d.	110	55	87	122	36	24	n.d.	n.d.	n.d.	n.d.
Acesulfame	10	1005	n.d.	n.d.	n.d.	n.d.	n.d.	2150	2545	3219	4570	1830	1268	285	17	n.d.	n.d.

Date of experiment 19.01.2011

Parameter [ng/l]	LOQ	CUV1	CNP1	CNP2	CNP3	CNP4	CNP	CNF1	CNF2	CNF3	CNF4	CAF2	CAK2/1	CAK2/2	CAK2/3	CAK2/4	CAAK2
1H-Benzotriazole	10	980	203	277	355	545	344	1805	2280	2766	3733	1155	19	n.d.	n.d.	n.d.	n.d.
Dimethylsufamide (DMS)	10	317	n.d.	n.d.	n.d.	14	n.d.	665	925	1057	1591	337	312	314	325	314	299
Metformin hydrochloride	10	400	18	29	37	70	37	3755	4940	5318	8529	420	1299	750	353	127	54
Methyl-Benzotriazole	10	1035	105	154	199	320	190	2090	2700	3421	4732	1145	16	n.d.	n.d.	n.d.	n.d.
Diglyme	10	18	24	n.d.	n.d.	n.d.	n.d.	65	35	17	177	27	60	10	n.d.	n.d.	n.d.
Desphenylchloridazone (DPC)	10	387	38	32	41	64	40	960	1185	1545	1811	378	66	n.d.	n.d.	n.d.	12
Caffeine	10	995	n.d.	12	n.d.	11	n.d.	2120	2685	3363	5178	1125	99	n.d.	31	23	n.d.
Isoproturon	10	109	n.d.	n.d.	n.d.	n.d.	n.d.	240	320	397	564	118	13	n.d.	n.d.	n.d.	n.d.
Primidone	10	1335	n.d.	n.d.	n.d.	n.d.	n.d.	2840	3595	4591	6683	1345	509	16	10	n.d.	n.d.
Terbutaline sulphate	10	950	n.d.	n.d.	n.d.	n.d.	n.d.	1995	2615	3289	4767	1295	42	n.d.	n.d.	n.d.	n.d.
Diuron	10	n.d.	n.d.	n.d.	n.d.	n.d.	n.d.	20	30	41	69	n.d.	n.d.	n.d.	n.d.	n.d.	n.d.
Carbamazepine	10	136	n.d.	n.d.	n.d.	n.d.	n.d.	300	390	481	670	140	17	n.d.	n.d.	n.d.	n.d.
Sulfamethoxazole	10	672	n.d.	n.d.	n.d.	n.d.	n.d.	1430	1835	2321	3286	1080	310	n.d.	n.d.	n.d.	n.d.
Propanolol	10	835	n.d.	n.d.	n.d.	n.d.	n.d.	1740	2380	2812	4241	1035	21	n.d.	n.d.	n.d.	n.d.
Diatrizoic acid	10	895	n.d.	n.d.	n.d.	n.d.	n.d.	1765	2330	2968	4195	1160	1022	571	234	35	n.d.
Iopamidole	10	18	n.d.	n.d.	n.d.	n.d.	n.d.	65	60	72	127	37	13	n.d.	n.d.	n.d.	n.d.
Acesulfame	10	1055	n.d.	n.d.	n.d.	n.d.	n.d.	2075	2900	3270	4802	1635	1447	271	18	n.d.	n.d.

Date of experiment 09.03.2011

Parameter [ng/l]	LOQ	CUV1	CNP1	CNP2	CNP3	CNP4	CNP	CNF1	CNF2	CNF3	CNF4	CAF2	CAK2/1	CAK2/2	CAK2/3	CAK2/4	CAAK2
1H-Benzotriazole	10	1083	260	323	419	591	410	2043	2337	3119	3999	1158	862	n.d.	n.d.	n.d.	n.d.
Dimethylsufamide (DMS)	10	391	14	15	15	23	19	854	1004	1403	1806	387	359	368	238	95	57
Metformin hydrochloride	10	3170	105	111	154	208	142	7737	10232	13369	20598	3580	3327	2121	1611	1007	631
Methyl-Benzotriazole	10	1064	121	156	212	315	196	2149	3114	3557	4995	1083	827	n.d.	n.d.	n.d.	n.d.
Diglyme	10	n.d.	n.d.	n.d.	n.d.	n.d.	n.d.	11	13	20	30	n.d.	n.d.	n.d.	n.d.	n.d.	n.d.
Desphenylchloridazone (DPC)	10	657	34	53	57	94	59	1265	1511	2125	2699	555	447	n.d.	n.d.	n.d.	n.d.
Caffeine	10	995	n.d.	n.d.	15	n.d.	n.d.	2204	2737	3724	4929	1021	850	n.d.	n.d.	45	n.d.
Isoproturon	10	118	n.d.	n.d.	n.d.	n.d.	n.d.	248	305	417	536	126	100	n.d.	n.d.	n.d.	n.d.
Primidone	10	963	n.d.	n.d.	n.d.	n.d.	n.d.	1845	2126	2855	3478	968	816	n.d.	n.d.	n.d.	n.d.
Terbutaline sulphate	10	894	n.d.	n.d.	n.d.	n.d.	n.d.	1162	1448	2375	3145	845	718	n.d.	n.d.	n.d.	n.d.
Diuron	10	n.d.	n.d.	n.d.	n.d.	n.d.	n.d.	16	16	19	28	n.d.	n.d.	n.d.	n.d.	n.d.	n.d.
Carbamazepine	10	144	n.d.	n.d.	n.d.	n.d.	n.d.	326	398	551	719	151	122	n.d.	n.d.	n.d.	n.d.
Sulfamethoxazole	10	1119	n.d.	n.d.	n.d.	n.d.	n.d.	1321	1566	2205	2948	1158	1000	n.d.	n.d.	n.d.	n.d.
Propanolol	10	1272	n.d.	n.d.	n.d.	13	n.d.	1803	2249	2830	4050	1288	958	n.d.	n.d.	n.d.	n.d.
Diatrizoic acid	10	1037	n.d.	n.d.	n.d.	n.d.	n.d.	1654	1928	2515	3426	1120	1073	n.d.	n.d.	n.d.	n.d.
Iopamidole	10	967	n.d.	n.d.	n.d.	n.d.	n.d.	1657	1806	2599	3620	984	1026	n.d.	n.d.	n.d.	n.d.
Acesulfame	10	1630	n.d.	n.d.	n.d.	n.d.	n.d.	2188	3007	3615	5155	1650	1520	n.d.	n.d.	n.d.	n.d.

Date of experiment 13.04.2011

Parameter [ng/l]	LOQ	CUV1	CNP1	CNP2	CNP3	CNP4	CNP	CNF1	CNF2	CNF3	CNF4	CAF2	CAK2/1	CAK2/2	CAK2/3	CAK2/4	CAAK2
1H-Benzotriazole	10	1190	241	380	479	707	430	2680	3445	3529	4650	1735	1395	n.d.	n.d.	n.d.	n.d.
Dimethylsufamide (DMS)	10	475	n.d.	n.d.	n.d.	n.d.	12	995	1420	1789	2607	530	535	496	444	422	451
Metformin hydrochloride	10	1895	42	48	90	190	71	6510	8375	8603	17900	2865	5488	4148	680	588	1064
Methyl-Benzotriazole	10	1350	113	204	212	314	277	2620	3600	3764	5960	1610	1472	n.d.	n.d.	n.d.	n.d.
Diglyme	10	23	n.d.	n.d.	n.d.	n.d.	n.d.	35	44	33	17	12	n.d.	50	38	20	n.d.
Desphenylchloridazone (DPC)	10	800	37	39	65	102	61	1030	1300	1745	2149	785	766	n.d.	n.d.	n.d.	n.d.
Caffeine	10	1310	15	30	27	14	65	2595	3040	4136	2791	1375	1279	n.d.	n.d.	12	35
Isoproturon	10	n.d.	n.d.	n.d.	n.d.	n.d.	n.d.	11	13	17	26	n.d.	n.d.	n.d.	n.d.	n.d.	n.d.
Primidone	10	1080	n.d.	n.d.	n.d.	n.d.	n.d.	2410	3045	4562	4926	1360	1071	26	n.d.	n.d.	n.d.
Terbutaline sulphate	10	440	n.d.	n.d.	n.d.	n.d.	n.d.	1070	1115	1766	2467	1005	822	n.d.	n.d.	n.d.	n.d.
Diuron	10	n.d.	n.d.	n.d.	n.d.	n.d.	n.d.	n.d.	n.d.	12	16	n.d.	n.d.	n.d.	n.d.	n.d.	n.d.
Carbamazepine	10	1270	n.d.	n.d.	n.d.	n.d.	n.d.	2630	3420	4624	6117	1490	1441	n.d.	n.d.	n.d.	n.d.
Sulfamethoxazole	10	660	n.d.	n.d.	n.d.	n.d.	n.d.	1510	1970	2532	3439	1610	1512	n.d.	n.d.	n.d.	n.d.
Propanolol	10	1320	n.d.	n.d.	n.d.	n.d.	n.d.	2225	2820	3450	5686	1860	2182	n.d.	n.d.	n.d.	n.d.
Diatrizoic acid	10	1085	n.d.	n.d.	n.d.	n.d.	n.d.	2215	2810	3682	4975	1665	1586	596	28	n.d.	n.d.
Iopamidole	10	620	n.d.	n.d.	n.d.	n.d.	n.d.	1835	1620	2365	3883	1005	754	132	n.d.	n.d.	n.d.
Acesulfame	10	1135	n.d.	n.d.	n.d.	n.d.	n.d.	2420	3520	4343	5347	1855	2019	115	n.d.	n.d.	n.d.

Group 3 Volatiles

Date of experiment 31.01.2011

Parameter [µg/l]	LOQ	CUV1	CNP1	CNP2	CNP3	CNP4	CNP	CNF1	CNF2	CNF3	CNF4	CAF2	CAK2/1	CAK2/2	CAK2/3	CAK2/4	CAAK2
Dichlormethane	0.01	5.89	4.13	5.88	6.18	7.18	5.7	6.88	7.22	7.73	8.29	5.62	3.82	2.88	1.84	0.92	0.41
MTBE	0.01	7.27	n.d.	n.d.	n.d.	0.09	0.06	15.07	18.89	22.5	29.11	7.17	4.23	3.29	2.92	1.22	0.58
ETBE	0.01	5.51	n.d.	n.d.	n.d.	n.d.	n.d.	14.04	16.85	19.93	29.09	5.16	3.12	1.42	0.73	0.06	n.d.
Trichloromethane	0.01	3.14	0.72	1.2	1.7	2.46	1.58	6	7.33	8.62	12.15	3.66	2.22	0.58	0.1	n.d.	n.d.
Benzene	0.01	4.87	0.42	0.71	1.06	1.57	0.86	9.88	12	13.8	20.25	4.28	0.35	n.d.	0.02	n.d.	n.d.
1,2-Dichlorethane	0.01	3.8	1.55	2.47	2.91	4.13	2.69	5.79	6.61	7.24	9.36	3.91	1.91	0.4	0.04	n.d.	n.d.
Trichloroethylene	0.01	0.81	0.26	0.36	0.41	0.59	0.39	1.29	1.55	1.77	2.44	1.72	0.27	n.d.	n.d.	n.d.	n.d.
Tetrachloroethylene	0.01	0.3	n.d.	n.d.	n.d.	n.d.	n.d.	0.49	0.69	0.83	1.26	0.57	n.d.	n.d.	n.d.	n.d.	n.d.

Date of experiment 02.02.2011

Parameter [µg/l]	LOQ	CUV1	CNP1	CNP2	CNP3	CNP4	CNP	CNF1	CNF2	CNF3	CNF4	CAF2	CAK2/1	CAK2/2	CAK2/3	CAK2/4	CAAK2
Dichlormethane	0.01	2.86	2.19	2.23	2.65	3.08	2.72	2.79	3.33	3.15	3.25	3.01	2.71	2.25	1.88	1.67	0.9
MTBE	0.01	4.97	n.d.	n.d.	n.d.	n.d.	n.d.	7.42	11.58	12.73	15.51	5.17	3.17	2.08	1.69	1.92	0.69
ETBE	0.01	4.65	n.d.	n.d.	n.d.	n.d.	n.d.	9.4	13.04	14.49	22.04	4.45	3.28	1.29	0.51	0.3	n.d.
Trichloromethane	0.01	3.16	0.86	1.18	1.73	2.66	1.71	5.46	7.08	7.96	11.46	3.51	2.5	0.63	0.12	n.d.	n.d.
Benzene	0.01	4.11	0.45	0.55	0.86	1.39	0.82	7.55	9.44	10.6	15.3	4.24	0.49	n.d.	n.d.	n.d.	n.d.
1,2-Dichlorethane	0.01	3.89	1.87	2.22	3.15	4.5	3.09	5.38	6.19	6.38	8.54	4.31	2.71	0.55	0.07	n.d.	n.d.
Trichloroethylene	0.01	1.17	0.38	0.47	0.63	0.86	0.61	1.91	2.36	2.61	3.61	1.64	0.3	n.d.	n.d.	n.d.	n.d.
Tetrachloroethylene	0.01	0.25	n.d.	n.d.	n.d.	n.d.	n.d.	0.5	0.67	0.8	1.22	0.5	n.d.	n.d.	n.d.	n.d.	n.d.

Date of experiment 16.03.2011

Parameter [µg/l]	LOQ	CUV1	CNP1	CNP2	CNP3	CNP4	CNP	CNF1	CNF2	CNF3	CNF4	CAF2	CAK2/1	CAK2/2	CAK2/3	CAK2/4	CAAK2
Dichlormethane	0.01	6.45	4.34	4.93	4.4	4.41	4.8	6.68	5.96	6.7	6.63	4.99	4.76	1.99	0.46	0.86	0.94
MTBE	0.01	5.21	n.d.	0.01	0.01	0.02	0.01	10.6	11.7	16.3	19.8	4.6	3.66	0.65	n.d.	n.d.	n.d.
ETBE	0.01	7.76	n.d.	n.d.	n.d.	n.d.	n.d.	16.6	18.3	26.9	33.8	7.11	6.12	n.d.	n.d.	n.d.	n.d.
Trichloromethane	0.01	2.97	0.7	0.937	1.01	1.37	1.03	4.95	5.28	7.21	8.79	2.34	2.36	n.d.	n.d.	n.d.	n.d.
Benzene-d6	0.01	0.64	0.09	0.11	0.12	0.12	0.12	0.68	0.64	0.68	0.73	0.79	0.91	n.d.	n.d.	n.d.	n.d.
Benzene	0.01	0.89	0.12	0.16	0.17	0.13	0.16	0.91	0.84	0.9	0.93	1.1	1.28	n.d.	n.d.	n.d.	n.d.
1,2-Dichlorethane	0.01	6.36	2.56	3.32	3.56	4.6	3.5	8.24	8.23	10.3	11.7	5.16	4.89	n.d.	n.d.	n.d.	n.d.
TAME	0.01	8.28	n.d	n.d	n.d	n.d	0.01	18	19.6	29	37.1	7.57	6.4	0.03	n.d.	n.d.	n.d.
Trichloroethylene	0.01	1.26	0.35	0.42	0.29	0.38	0.42	1.8	1.99	2.4	3.03	1.02	0.93	n.d.	n.d.	n.d.	n.d.
Dioxan	0.1	8.21	n.d.	n.d.	n.d.	n.d	n.d.	21.9	24.8	38.8	58.3	8.02	5.443	4.69	n.d.	n.d.	n.d.
Tetrachloroethylene	0.01	0.45	0.03	0.03	0.04	0.04	0.04	0.84	0.96	1.23	1.66	0.49	0.38	n.d.	n.d.	n.d.	n.d.

Date of experiment 20.04.2011

Parameter [µg/l]	LOQ	CUV1	CNP1	CNP2	CNP3	CNP4	CNP	CNF1	CNF2	CNF3	CNF4	CAF2	CAK2/1	CAK2/2	CAK2/3	CAK2/4	CAAK2
Dichlormethane	0.01	3.5	2.71	2.22	2.46	3.3	2.6	2.54	2.29	2.35	2.86	2.82	1.73	2.54	2.53	2.05	1.42
MTBE	0.01	7.54	0.01	0.01	0.01	0.02	0.01	9.64	10.3	14.2	21.3	5.72	3.71	3.26	1.84	0.63	0.25
ETBE	0.01	5.37	n.d.	n.d.	n.d.	0.01	0.01	15.2	17	23.1	36.6	8.39	6.33	3.38	0.47	n.d.	n.d.
Trichloromethane	0.01	1.58	0.4	0.4	0.48	0.76	0.49	1.87	2.12	2.68	3.92	2.09	1.58	0.79	0.05	0.04	n.d.
1,2-Dichlorethane	0.01	2.13	1.02	0.86	1.06	1.63	1.12	2.56	2.76	3.25	4.6	3.08	2.21	1.3	0.09	0.15	n.d.
TAME	0.01	5.07	0.01	n.d.	n.d.	n.d.	n.d.	14.7	16.6	22.7	36.8	8.14	6.07	2.34	0.13	n.d.	n.d.
Trichloroethylene	0.01	0.44	0.16	0.16	0.16	0.25	0.17	0.73	0.87	1.09	1.45	0.86	0.65	n.d.	n.d.	n.d.	n.d.
Dioxan	0.1	5.31	n.d.	n.d.	n.d.	0.1	0.15	19.4	25.1	40.1	68.6	11	17.3	6.48	5.86	5.48	5.48
Tetrachloroethylene	0.01	0.31	0.02	0.02	0.03	0.05	0.03	0.28	0.34	0.42	0.63	0.2	0.15	n.d.	n.d.	n.d.	n.d.

Group 3 Aminopolycarboxylic acids

Date of experiment 02.02.2011

Parameter [µg/l]	LOQ	CAF1	CUV1	CNP1	CNP2	CNP3	CNP4	CNP	CNF1	CNF2	CNF3	CNF4	CAF2	CAK2/1	CAK2/2	CAK2/3	CAK2/4	CAAK2
EDTA	0.5	-	11	n.d.	n.d.	n.d.	n.d.	n.d.	38	46	54	83	11	9.9	8.6	6.1	4.3	2.8
DTPA	1	-	7.6	n.d.	n.d.	n.d.	n.d.	n.d.	30	35	44	56	6.2	7.8	3.1	1.4	n.d.	n.d.

Date of experiment 16.03.2011

Parameter [µg/l]	LOQ	CAF1	CUV1	CNP1	CNP2	CNP3	CNP4	CNP	CNF1	CNF2	CNF3	CNF4	CAF2	CAK2/1	CAK2/2	CAK2/3	CAK2/4	CAAK2
EDTA	0.5	-	13	-	-	-	n.d.	-	-	-	-	71	15	15	3.5	n.d.	n.d.	n.d.
DTPA	1	-	9.1	-	-	-	n.d.	-	-	-	-	47	9.4	5.3	n.d.	n.d.	n.d.	n.d.

Date of experiment 20.04.2011

Parameter [µg/l]	LOQ	CAF1	CUV1	CNP1	CNP2	CNP3	CNP4	CNP	CNF1	CNF2	CNF3	CNF4	CAF2	CAK2/1	CAK2/2	CAK2/3	CAK2/4	CAAK2
EDTA	0.5	-	-	-	-	-	-	-	-	-	-	-	25	14	10	4.8	1.5	0.5
DTPA	1	-	-	-	-	-	-	-	-	-	-	-	30	14	5	n.d.	n.d.	n.d.

Nitrosamines

Date of experiment 02.02.2011

Parameter [ng/l]	LOQ	CAF1	CUV1	CNP1	CNP2	CNP3	CNP4	CNP	CNF1	CNF2	CNF3	CNF4	CAF2	CAK2/1	CAK2/2	CAK2/3	CAK2/4	CAAK2
NDMA	1	22	14	5.8	8.8.	11	15	11	21	23	28	35	23	23	19	15	12	6.9
NMOR	1	120	36	n.d.	1.4	2.3	3.8	1.8	73	93	120	180	120	98	52	17	5.8	2.7

Date of experiment 20.04.2011

Parameter [ng/l]	LOQ	CAF1	CUV1	CNP1	CNP2	CNP3	CNP4	CNP	CNF1	CNF2	CNF3	CNF4	CAF2	CAK2/1	CAK2/2	CAK2/3	CAK2/4	CAAK2
NDMA	1	20	14	7.3	8.3	11	13	9.1	19	26	33	40	25	26	24	21	14	10
NMOR	1	25	12	n.d.	n.d.	n.d.	1.3	n.d.	20	27	39	56	30	31	16	5.6	1.1	n.d.

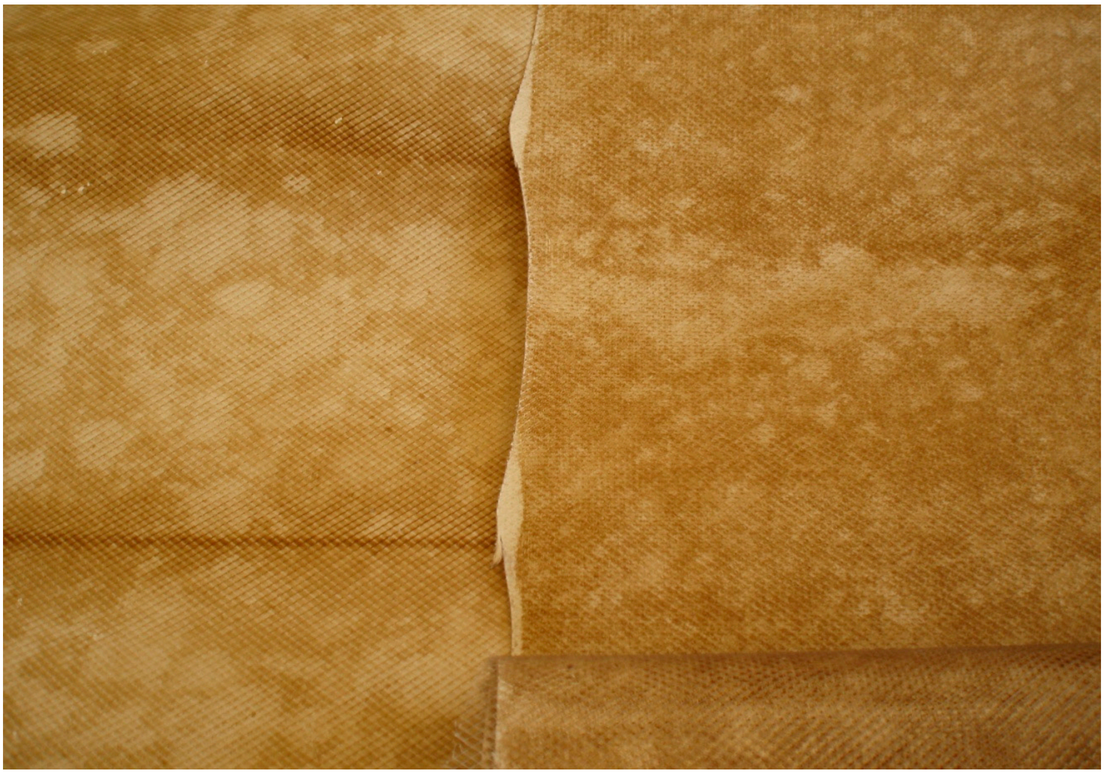

Figure S1. Results of membrane autopsy.

Membrane during autopsy, left side: leading element, right side: last element.

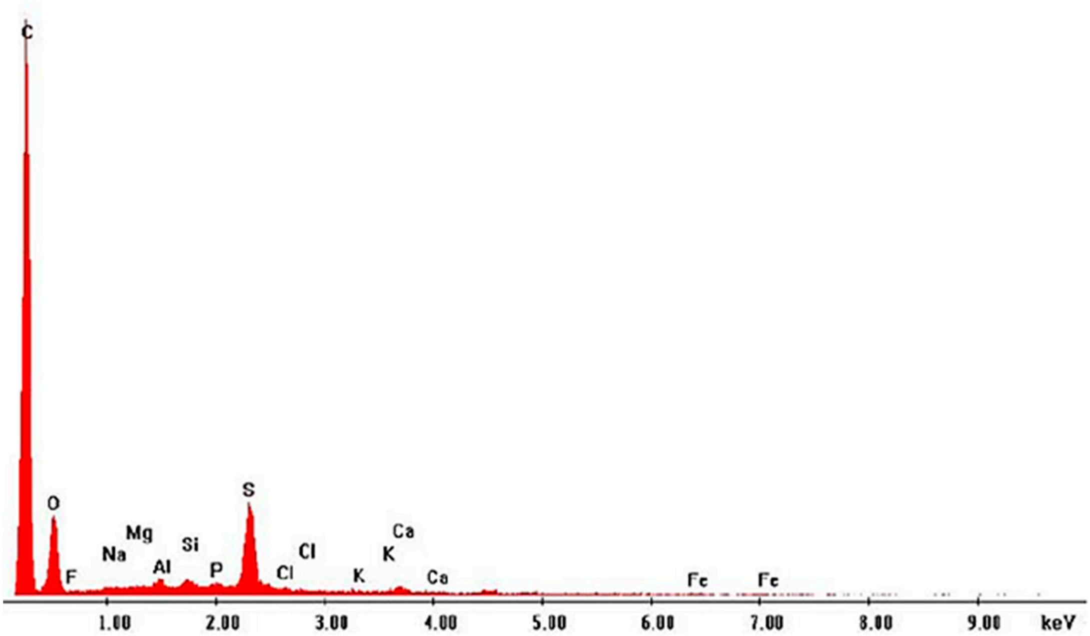

EDX analysis of leading element with slightly scaling.

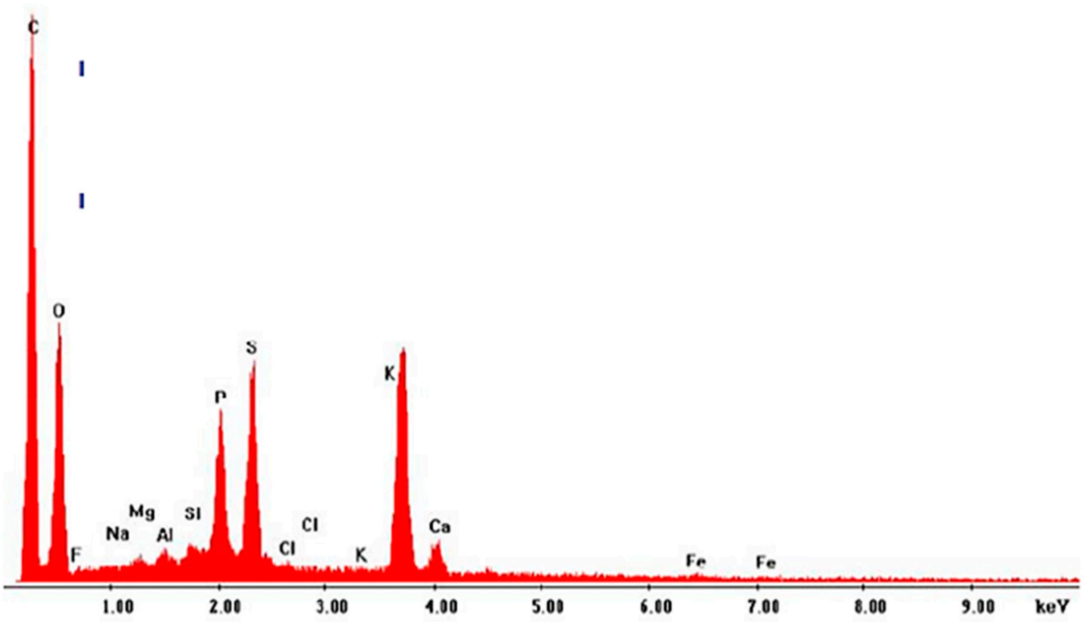

EDX analysis of last element with calcium phosphate-calcite and weak silicate scaling.

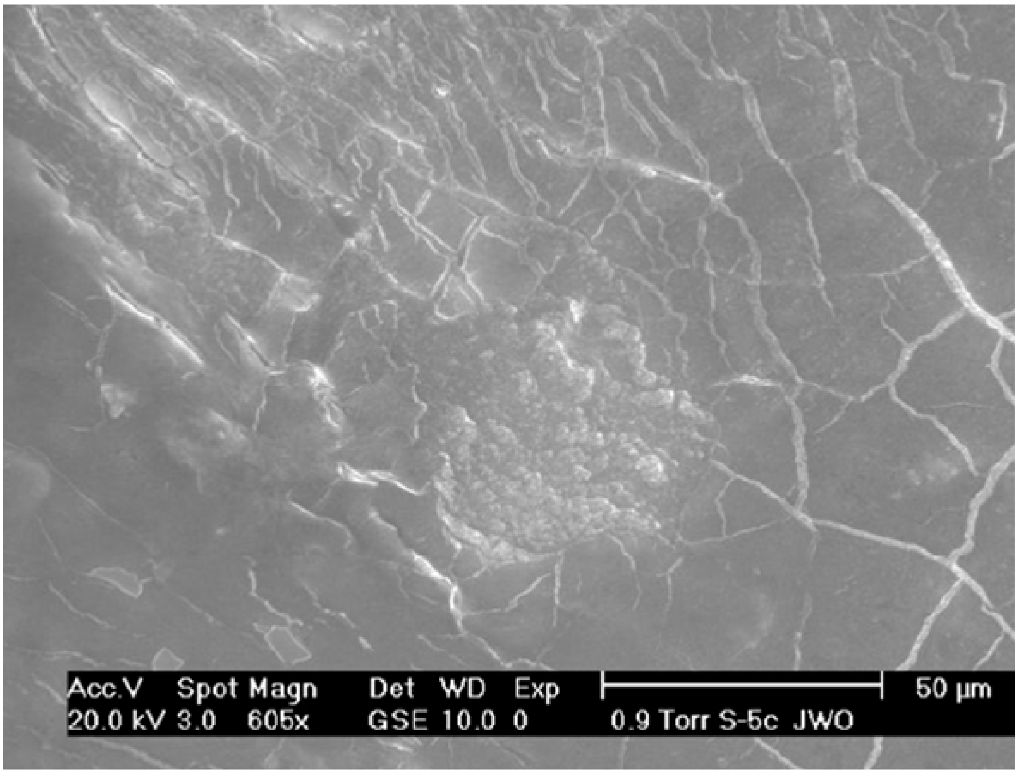

ESEM analysis of leading element (dried surface with slightly scaling).

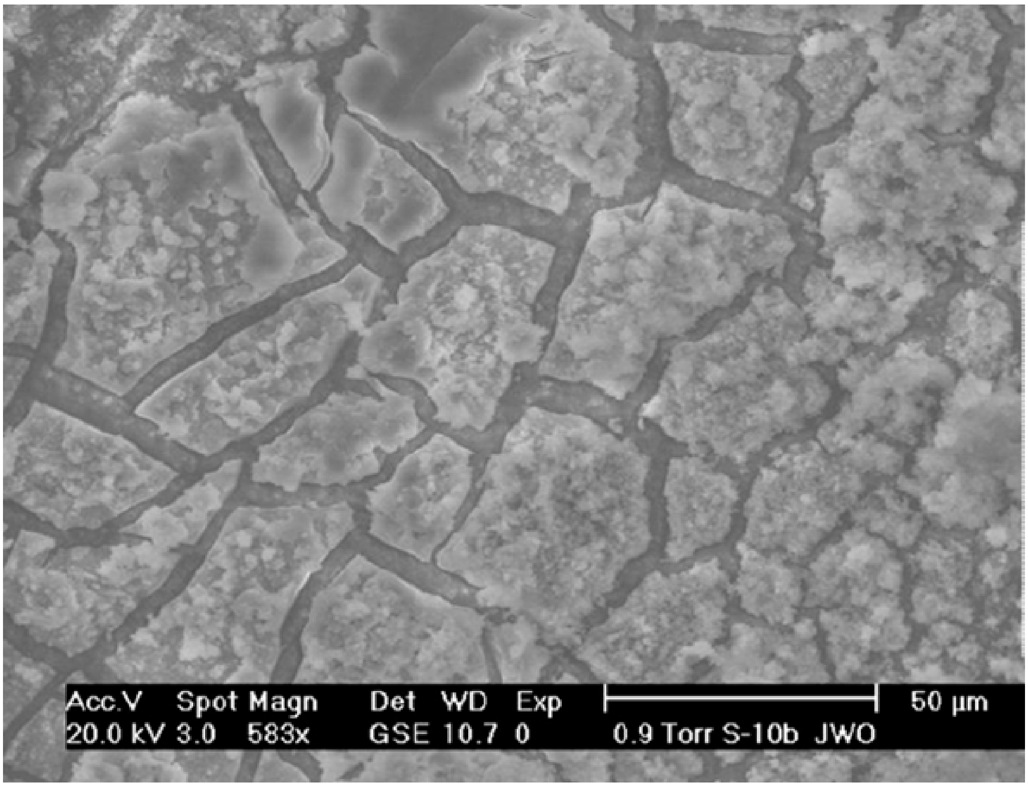

ESEM analysis of last element (dried surface with scaling).
